# The Association Between Indoor Air Pollutants and Brain Structure Indicators Using eTIV-Adjusted and Unadjusted Models: A Study in Seoul and Incheon

**DOI:** 10.3390/brainsci15080868

**Published:** 2025-08-14

**Authors:** Sun-Min An, Ho-Hyun Kim

**Affiliations:** 1 Department of Research, Institute for Living and Industrial Environment, Seokyeong University, Seoul 02173, Republic of Korea; sunmin4314@naver.com; 2 Department of Environmental and Chemical Engineering, Seokyeong University, Seoul 02173, Republic of Korea

**Keywords:** indoor air pollution, particulate matter (PM_10_; PM_2.5_), brain, estimated total intracranial volume (eTIV), Internet of Things (IoT)

## Abstract

Background/Objectives: As older adults spend increasing amounts of time indoors, concerns are rising about the neurological effects of indoor air pollution. This study examined associations between indoor air pollutants and structural brain changes in community-dwelling older adults in Seoul and Incheon, South Korea. A purposive sample of 23 individuals aged ≥65 years was recruited. Internet of Things (IoT)-based devices were installed in participants’ homes to continuously monitor indoor concentrations of PM_10_, PM_2.5_, and CO_2_ for over two months. All participants underwent 3T brain magnetic resonance imaging (MRI), and brain structure metrics were analyzed using multiple linear regression models with and without adjustment for estimated total intracranial volume (eTIV). Hierarchical clustering was also performed based on exposure and neuroanatomical characteristics. Brain MRI indicators included cortical surface area, cortical thickness in six regions, and volumes of seven subcortical structures including the hippocampus and amygdala. Higher CO_2_ concentrations were significantly associated with lower hippocampal volumes in both hemispheres (left: −2.83, −0.88, −1.02 mm^3^; right: −3.29, −0.86, −0.99 mm^3^; *p* ≤ 0.05). Elevated PM_2.5_ levels were associated with reduced bilateral amygdala volume (−283.24 mm^3^ left; −292.37 mm^3^ right) and right hippocampal volume (−544.55 mm^3^; *p* ≤ 0.05). Cluster analysis showed that, before eTIV adjustment, Group C exhibited the lowest subcortical volumes. After adjustment, Group A showed the smallest cortical surface area, and Group D had the lowest subcortical volumes. These findings suggest that indoor air pollutants, including PM_10_, PM_2.5_, and CO_2_, may be associated with structural brain alterations in older adults, supporting the need for age-specific indoor air quality standards and residential monitoring systems.

## 1. Introduction

According to the World Health Organization (WHO), 90% of the world’s population reside in areas with air quality parameters exceeding WHO Air Quality Guidelines, and indoor air pollutants, including particulate matter (PM_10_), fine particulate matter (PM_2.5_), carbon dioxide (CO_2_), formaldehyde, and volatile organic compounds (VOCs), account for approximately 3.8 million deaths annually [1,2,3,4]. The indoor concentrations of some air pollutants are two to five times higher than their outdoor concentrations [5,6,7].

As the tendency to spend more time indoors increases due to changes in lifestyle, the health effects of indoor air pollutants are becoming more obvious [8,9]. Several studies have been conducted to investigate the effects of PM_10_ and PM_2.5_ on the occurrence of cerebropathies in Korea and other countries [10,11,12]. In addition, a cohort study in Taiwan indicated that a 4.34 µg/m^3^ increase in PM_2.5_ concentration was associated with a 138% increase in the incidence of Alzheimer’s disease [13]. Another study in the UK showed that a 2.0 µg/m^3^ increase in PM_2.5_ concentrations resulted in a 0.32% decrease in brain volume and increased the risk of cerebral infarction in individuals aged 60 years or older [14]. Nano-scale particles such as PM_10_ can easily be transported to the brain via the olfactory epithelium without crossing the blood–brain barrier, eventually triggering cardiovascular and cerebrovascular diseases [15,16]. According to the Lawrence Berkeley National Laboratory, an increase in indoor CO_2_ concentrations from 600 to 2500 ppm results in a decline in brain structure [17,18]. Studies on exposure to formaldehyde, which have been conducted predominantly using animals, have also shown that formaldehyde exposure negatively affects brain volume and white matter [19,20]. Exposure to VOCs has also been shown to induce neurological disorders, including cognitive changes, hallucinations, dizziness, and dementia, in humans [21]. Additionally, highly toxic BTEX compounds (benzene, toluene, ethylbenzene, and xylene) can cause neuropsychiatric changes, such as dementia, headaches, learning disabilities, and memory loss, and toluene exposure negatively affects white matter in the brain [22].

Vulnerable populations, particularly infants, older adults, and those with underlying medical conditions, spend considerably more time indoors than the general population [23]. They are therefore at a greater risk of exposure to indoor air pollutants and are more likely to suffer more severely from indoor air pollutant-related health issues, even when exposed to the same pollutant concentrations as the general population [24]. According to the National Institute of Environmental Research, Koreans spend an average of 20.66 h indoors daily, with 15.86 h of this time spent at home [25]. Thus, there is an urgent need to investigate the effects of indoor air quality on brain health.

Recent neuroimaging studies have highlighted the importance of adjusting for intracranial volume (ICV) as a covariate, given that individual differences in skull size can influence the outcomes of brain structure analyses [26,27,28]. For example, Canning et al. [29] explicitly controlled for ICV and age at the time of the scan in their MRI-based analysis of environmental exposure and cognitive function, demonstrating that ICV adjustment is systematically incorporated into statistical modeling in neuroimaging cohorts. Similarly, Thompson et al. [30] investigated whether MRI-derived brain volumes mediated the association between air pollution and dementia risk, implying that volumetric normalization for cranial size was embedded in their analytical framework. Following these approaches, the present study compared results before and after eTIV correction to enhance analytical precision and improve comparability with prior research. Although ICV and estimated total intracranial volume (eTIV) differ in calculation methods, eTIV derived from FreeSurfer software version 6.0 was used as a reliable proxy for ICV in this study [27,31]. This approach is expected to contribute to a more accurate understanding of the relationship between indoor air quality exposure and brain structure in older adults.

In this study, we examined the association between indoor air pollutant concentrations and brain-related indicators from the perspective of brain volume using indoor air quality data from residential spaces obtained using IoT devices. The findings of this study may serve as a basis for policy recommendations aimed at facilitating the prevention and management of brain diseases.

## 2. Materials and Methods

### 2.1. Study Design and Population

**Figure 1:**
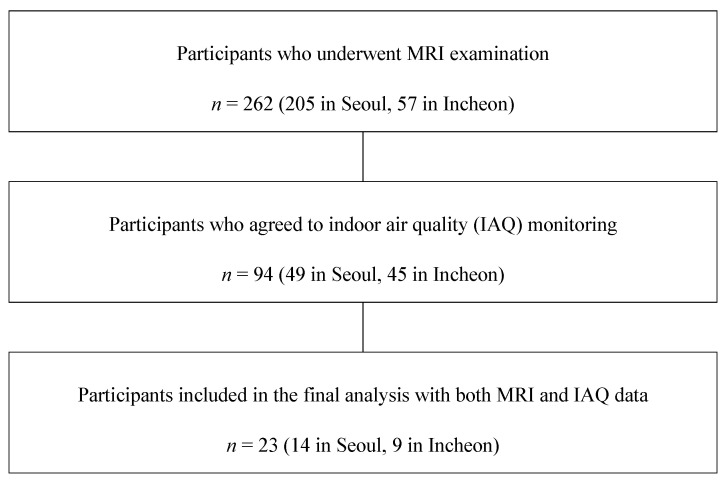
Flow diagram showing the process used to select study participants.

This pilot cross-sectional study was conducted as part of the Environmental Pollution-Induced Neurological Effects (EPINEF) project to explore the potential impact of environmental pollution on brain structure among community-dwelling older adults aged 65 years or older. Participants were initially recruited from two metropolitan areas in Korea (Seoul and Incheon) through voluntary participation in a brain magnetic resonance imaging (MRI) examination [32,33]. Among 262 individuals who underwent MRI (205 in Seoul and 57 in Incheon), 94 agreed to participate in additional indoor air quality (IAQ) monitoring [34,35] in accordance with the Ministry of Environment’s official test method for indoor air quality [36]. Of these, 23 participants (14 from Seoul and 9 from Incheon) who were able to install IoT-based air quality monitoring equipment in their homes for at least two months were included in the final analysis (Figure 1). As this was a pilot study, the final sample size was primarily determined based on feasibility. This study was approved by the Institutional Review Board of the Yonsei University Health System (Approval No. 4-2022-0418).

### 2.2. Measurement of Concentrations of Indoor Air Pollutants

In this study, we monitored three air pollutants, PM_10_, PM_2.5_, and CO_2_, using IoT measurement equipment (Smart Aircok; Aircok, Seoul, Republic of Korea) installed in the kitchens of the participants. The device used was certified as Grade 1 under the Ministry of Environment’s performance certification system for simplified measurement devices, pursuant to relevant regulations on indoor air quality management [37]. For PM, certification is based on comparison with reference instruments in terms of the coefficient of determination (R^2^), accuracy, precision, and repeatability. For CO_2_, Grade 1 certification requires a repeatability ≤ 10%, linearity ≤ 15%, and relative accuracy ≤ 30%, as defined in the official notification. In this study, only Grade-1-certified sensors that fully complied with these criteria were used, and thus, the measurements were considered valid and reliable according to national regulatory standards [37].

The kitchen was selected as the primary monitoring site based on prior studies identifying it as a major source of indoor air pollutants during cooking activities [38,39,40]. Although no official guidelines exist in Korea regarding indoor monitoring locations in residential settings, this approach is consistent with standard practice in similar studies. When installation in the kitchen was not feasible due to safety or structural constraints, the monitoring device was placed in the nearest adjacent room. The observation period varied by region: in Seoul, it was 4 April to 16 July 2023, and in Incheon, it was from 31 August 2023 to 1 February 2024. All measured data were collected in real time at 5 min intervals, and were averaged into 24 h (1 day) values before analysis.

### 2.3. Selection of Brain MRI Indicators

Brain MRI examinations of the 23 participants were performed using a Philips 3T Achieva MRI scanner (Philips Healthcare, Amsterdam, The Netherlands) at one of two University Hospitals: Severance Hospital, Yonsei University (Seoul) and Gil Medical Center, Gachon University (Incheon). The brain MRI was performed according to a standardized protocol, and to estimate cortical thickness, subcortical gray matter volume, and total brain volume, 3D T1 magnetization-prepared rapid acquisition gradient echo images (3D T1-weighted MPRAGE) were analyzed using FreeSurfer software version 6.0, which automatically analyzes brain MRI images. Finally, cortical surface area, cerebral cortex thickness (frontal lobe, parietal lobe, temporal lobe, occipital lobe, cingulate gyrus, and insular lobe), and subcortical structures (nucleus accumbens, amygdala, hippocampus, globus pallidus, putamen, caudate nucleus, and thalamus) were selected as brain MRI indicators.

### 2.4. Covariates

The covariates in this study included height, weight, total intracranial volume, sex, education level, past medical history (e.g., angina, hypertension, hyperlipidemia, and diabetes), age, and body mass index (BMI), and these were selected according to previous studies [41,42,43,44]. Data on these covariates were collected using questionnaires and physical examinations. Education level was categorized as follows: never attended school, elementary school dropout, elementary school graduate, middle school dropout, middle school graduate, high school dropout, high school graduate, technical/vocational school graduate, university dropout, university graduate, and postgraduate degree or higher. With respect to age, the participants were categorized as older adults aged ≥75 years and those aged <75 years, and according to BMI, as individuals with BMI ≥ 25 and those with BMI < 25. Height, weight, and total intracranial volume were not categorized given that they are continuous variables.

### 2.5. Multiple Regression Model

Statistical analyses were performed using indoor air quality data collected via the IoT equipment and brain MRI indicators. Before analysis, indoor air quality data were refined into four variables (minimum, average, maximum, and geometric mean), which were then used alongside MRI survey data to quantify the impact of environmental factors on brain structure indicators using multiple regression models. Basically, a multiple regression model is a statistical model that indicates the relationship between a dependent and an independent variable, assuming that the dependent variable is linearly influenced by more than one independent variable [45]. The multiple regression model used was:

y_i_ = β_0_ + β_1_x_1i_ + β_2_x_2i_ + … + β_p_x_pi_ + ε_i_ → [i = 1, 2, …, n] (1)

where β_0_, …, β_p_ represent regression coefficients; x_1i_, …, x_pi_ represent explanatory variables (independent variables); y_i_ represents response variables (dependent variables); and ε_i_ represents the error term.

In the multiple regression models (Models 1–4), we corrected for the effects of environmental factors, as well as those of personal characteristics (e.g., age, gender, BMI, and education level) that could affect MRI variables. Specifically, the following models were applied:

- Model 1 assessed the association between each brain MRI feature and a single continuous environmental variable, adjusting for personal characteristics.
- Model 2 extended Model 1 by additionally adjusting for total intracranial volume.
- Model 3 was identical to Model 1, except that the continuous environmental variable was replaced with a dichotomous variable derived using its median as a cut-off.
- Model 4 extended Model 3 by also adjusting for total intracranial volume.

Statistical analyses were further performed using two-tailed tests, with the level of significance (alpha) set at *p* = 0.05.

### 2.6. Hierarchical Clustering

To investigate the effects of environmental variables on brain structural features derived from MRI data, we applied hierarchical clustering after adjusting for individual-level covariates. First, we corrected for the effects of individual characteristics (such as age, sex, and education) on these MRI-derived features using multiple linear regression models. The residuals from these models, representing variance unexplained by the covariates, were used as input for the clustering analysis to minimize the influence of individual-level confounders.

In addition, given the importance of total intracranial volume adjustment in neuroimaging analyses, we further accounted for total intracranial volume by including it as an additional covariate in the regression models. Residuals were then re-derived from models including both individual characteristics and total intracranial volume, allowing for clustering based on brain features independent of these effects.

After this correction and standardization process (to adjust for unit differences across features), we conducted hierarchical clustering using Euclidean distance and Ward’s linkage method. The optimal number of clusters was determined based on silhouette scores. All statistical analyses were performed using R software (version 4.3.1) [46].

## 3. Results

### 3.1. Characteristics of Study Participants

The mean age, height, and weight of the 23 study participants were 75 years, 156.2 cm, and 61.2 kg, respectively. The sample included 16 females and 7 males; 13 (56.5%) had a BMI below 25 and 18 (78.3%) were educated above high school level. A total of 19 participants had no history of angina (82.6%) and 18 had no history of diabetes (78.3%). However, 17 (73.9%) and 14 (60.9%) had a history of hypertension and hyperlipidemia (60.9%), respectively, indicating that more than half of the research participants had an underlying disease (Table 1).

### 3.2. Indoor Air Pollutants and MRI Results

Combining the Seoul and Incheon cohorts, the average indoor concentrations of PM_2.5_ (17.99 µg/m^3^), PM_10_ (24.07 µg/m^3^), and CO_2_ (791.59 ppm) were all below the respective thresholds for facilities for vulnerable populations specified in the Enforcement Decree of the Indoor Air Quality Control Act [47], which are 35 µg/m^3^ for PM_2.5_, 75 µg/m^3^ for PM_10_, and 1000 ppm for CO_2_ (Table 2 and Table S1).

Compared to the WHO’s 24 h mean air quality guidelines, the PM_2.5_ concentration exceeded the limit of 15 µg/m^3^, while the PM_10_ concentration remained below the 45 µg/m^3^ threshold [2]. WHO does not provide a health-based guideline for CO_2_, noting instead that it is often used as a surrogate marker for ventilation adequacy. According to Mendell et al. [48], the most commonly used indoor CO_2_ threshold is 1000 ppm, which serves as a practical indicator of acceptable indoor air quality.

These values were derived from indoor measurements conducted in participant’s homes during the designated monitoring period. Of the MRI variables (combining the Seoul and Incheon cohorts), the mean total cerebral volume was 1,530,571 mm^3^ and the mean cerebral surface areas of the left and right hemispheres were 75,784 mm^2^ and 75,876 mm^2^, respectively. Additionally, the mean thickness of the entire cerebral cortex was 2.35 mm. The average volumes of the subcortical structures, including the nucleus accumbens, amygdala, hippocampus, globus pallidus, putamen, caudate nucleus, and thalamus, were 365 mm^3^, 1360 mm^3^, 3593 mm^3^, 1776 mm^3^, 4107 mm^3^, 2999 mm^3^, and 6151 mm^3^, respectively (Table S2).

### 3.3. Multiple Regression Analysis Results

Model 1, with the new environmental variables as independent variables, showed that left hemisphere hippocampal volume (min est: −2.83 mm^3^, *p* = 0.05; mean est: −0.88 mm^3^, *p* = 0.05; gmean est: −1.02 mm^3^, *p* < 0.05) and right hemisphere hippocampal volume (min est: −3.29 mm^3^, *p* < 0.05; mean est: −0.86 mm^3^, *p* < 0.05; gmean est: −0.99 mm^3^, *p* < 0.05) were negatively correlated with higher CO_2_ concentrations (Table 3 and Table S3). Model 2 showed results similar to those obtained for Model 1, despite the addition of individual cerebral volumes (Table 3 and Table S4). For Model 3, in which categorical variables were used based on the median values of the new environmental variables and were included as independent variables, left hemisphere amygdala volume (mean est: −283.24 mm^3^, *p* < 0.05), right hemisphere amygdala volume (mean est: −292.37 mm^3^, *p* < 0.05), and right hemisphere hippocampal volume (mean est: −544.55 mm^3^, *p* = 0.05) showed negative correlations with higher PM_2.5_ concentrations (Table 4 and Table S5). Model 4 showed results similar to those obtained for Model 3 despite the consideration of individual cerebral volumes (Table 4 and Table S6).

### 3.4. Hierarchical Clustering Results

**Figure 2:**
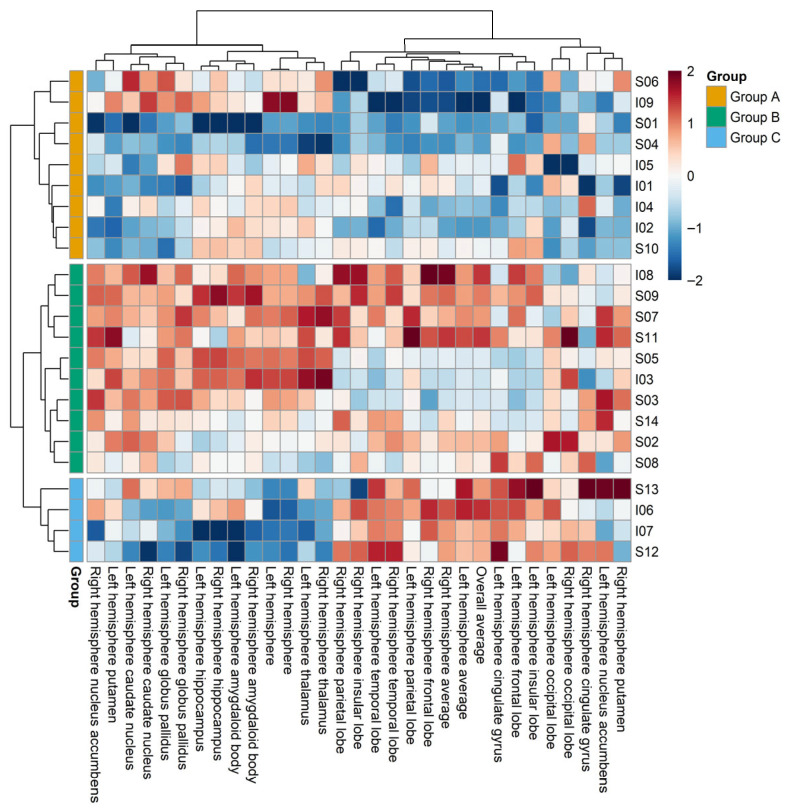
Heatmap showing hierarchical clustering results without correction for intracranial volume.

Hierarchical clustering was performed after removing the effects of 10 individual characteristics (e.g., height, weight, gender, and education level). This analysis identified three distinct clusters—Groups A, B, and C—which were characterized by different combinations of neuroanatomical features, including cortical thickness, cortical surface area, and subcortical volume (Figure 2). Of the 23 participants, Groups A, B, and C comprised 9 (39.1%), 10 (43.5%), and 4 (17.4%) individuals, respectively.

When comparing MRI-derived metrics across groups, Group A exhibited the lowest mean cortical thickness (mean z-score: Group A = −0.93, Group B = 0.46, Group C = 0.94), whereas Group C showed the lowest cortical surface area (left hemisphere: Group A = −0.08, Group B = 0.64, Group C = −1.43; right hemisphere: Group A = −0.04, Group B = 0.62, Group C = −1.45). Subcortical volumes also varied by region. Group C generally exhibited lower volumes across multiple subcortical structures. Regarding environmental exposure, Group C was characterized by the highest average concentrations of indoor air pollutants, including PM_2.5_, PM_10_, and CO_2_ (Table S7).

**Figure 3:**
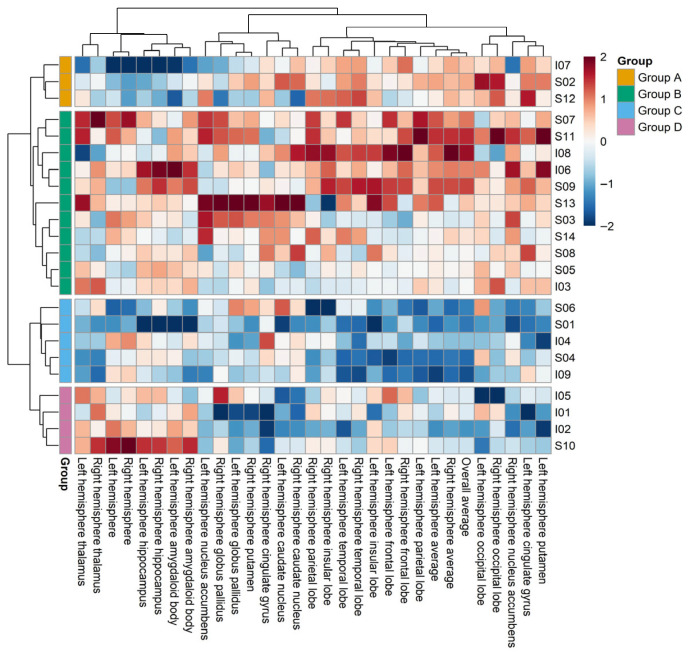
Heatmap showing hierarchical clustering with correction for intracranial volume.

A second clustering analysis was conducted after additionally adjusting for total intracranial volume (eTIV), in combination with the original 10 individual characteristics. This resulted in four groups—Groups A, B, C, and D—each reflecting distinct profiles driven primarily by variations in cortical thickness, subcortical volume, cortical surface area, and a combination of surface area and volume, respectively (Figure 3). The number of participants in Groups A, B, C, and D were 3 (13.0%), 11 (47.8%), 5 (21.7%), and 4 (17.4%), respectively.

Group C showed the lowest mean cerebral cortex thickness (Group A = 0.57, Group B = 0.66, Group C = −1.37, Group D = −0.52), whereas Group A presented the lowest cortical surface area (left hemisphere: Group A = −1.24, Group B = 0.28, Group C = −0.41, Group D = 0.69; right hemisphere: Group A = −1.47, Group B = 0.25, Group C = −0.25, Group D = 0.73). Regarding subcortical structures, the volumes of the nucleus accumbens, hippocampus, and caudate nucleus were lowest in Groups D, A, and C, respectively. The volumes of the amygdala, globus pallidus, and putamen showed varied trends depending on environmental exposure levels. Notably, Groups A and D exhibited higher average exposures to PM_10_ and CO_2_ compared to the other groups (Table S7).

Overall, distinct characteristics were observed across clusters when considering both environmental exposure levels and neuroanatomical patterns. Before ICV correction, Group C exhibited the highest concentrations of indoor air pollutants and generally lower subcortical volumes. After ICV correction, Groups A and D showed relatively higher levels of PM_10_ and CO_2_, which were associated with reduced cortical surface area in Group A and decreased subcortical volumes in Group D.

These trends are summarized descriptively in the main text, and detailed values for pollutant concentrations and brain structure metrics are provided in Supplementary Tables S7 and S8.

## 4. Discussion

In this study, we monitored the concentrations of indoor air pollutants in the residential spaces of 23 individuals aged 65 years and older, and thereafter investigated the effects of these indoor air pollutants on brain structure before and after brain volume correction using a multiple regression model and hierarchical clustering.

Higher PM_2.5_ and PM_10_ concentrations were negatively associated with brain-related indicators. Specifically, PM_2.5_ had an effect on left and right hemisphere hippocampal volume (*p* < 0.05), and CO_2_ was found to affect left hemisphere amygdala volume, right hemisphere amygdala volume, and right hemisphere hippocampal volume (*p* < 0.05).

These relationships between indoor air pollutant levels and brain structure are consistent with those reported in previous studies. Balboni et al. [49] found that exposure to nitrogen dioxide, PM_10_, and PM_2.5_ decreased the hippocampal volume in adults, and particularly, under high PM_2.5_ concentrations, hippocampal volume decreased further. Similarly, Cho et al. [33] observed that a 10 µg/m^3^ increase in PM_10_ concentration was associated with a 55.4 mm^3^ decrease in hippocampal volume. The hippocampus is one of the areas of the brain responsible for learning and remembering new information, and a decrease in its volume causes a deterioration in cognitive function, leading to neurological disorders, such as dementia [50]. Alzheimer’s disease, a neurodegenerative condition, has also been associated with exposure to air pollutants, such as nitrogen dioxide and PM_10_, and the hippocampus is the first part of the brain that reportedly shrinks as symptoms of Alzheimer’s disease progress [11,51].

Previous studies on the correlation between CO_2_ concentration and brain structure have shown that high CO_2_ concentrations affect the functioning of brain structures, such as brain blood vessels and the amygdala [52,53,54,55]. In a study involving rats [56], long-term exposure to CO_2_ not only influenced brain structures (e.g., hippocampus, prefrontal cortex, and amygdala), but it also lowered cognitive and decision-making functions [57,58]. In a cohort study involving 24 participants, Allen et al. [59] reported an association between higher indoor CO_2_ concentrations and a lower cognitive function. The mean concentration of CO_2_ reported in Allen’s study was 500~1400 ppm, similar to that obtained in the current study.

Recent advances in automated brain imaging techniques have contributed considerably to the growing body of knowledge about the brain’s structure. Brain structure varies according to demographic variables, such as sex, region, age, height, and weight [60,61,62]. In the current study, a comparison of individual cerebral volumes pre- and post-correction (estimated total intracranial volume, eTIV) in line with brain imaging techniques indicated no statistical differences with respect to these variables. Barnes et al. [60] reported a negative association between cortical thickness and intracranial volume; however, this association disappeared after correcting for age and sex [61]. Individual cerebral volumes and eTIVs are affected by demographic variables [27,62,63,64,65].

In the current study, hierarchical clustering was conducted to examine the relationship between brain structure and environmental factors by clustering research targets based on MRI variables. Hierarchical clustering is a maximum similarity data grouping method, and its strength lies in its ability to facilitate the identification of associations between groups [66]. Based on our results without correcting for cerebral volume, three groups relating to cerebral cortex thickness (Group A), cerebral surface area (Group B), and cerebral volume (Group C), were identified. However, after cerebral volume was corrected for, hierarchical clustering resulted in four groups, Groups A, B, C, and D, relating to cerebral cortex thickness, cerebral surface area, cerebral volume, and cerebral surface area and cerebral volume, respectively. In the clustering results that were not corrected for cerebral volume, Group C had the lowest cerebral surface area and was exposed to higher CO_2_ concentrations than the other groups. This observation implies that environmental factors can markedly influence brain surface area. In the clustering results that were corrected for cerebral volume, Groups C and A had the lowest cerebral cortex thickness and cerebral surface area, respectively. The smallest cerebral surface area and the higher CO_2_ concentration observed in Group A relative to the other groups implies that CO_2_ may affect brain structure. Consistent with this conclusion, Gellhorn [67] suggested that CO_2_ destroys neurons in the cortex and hypothalamus, and noted that long-term exposure to high concentrations of CO_2_ can temporarily pause cerebral cortex function.

This study suggests that indoor air pollutants such as PM_2.5_, PM_10_, and CO_2_ may negatively affect brain structure in older adults, consistent with previous epidemiological and experimental findings [17,18,68,69]. While this study focused on indoor air quality, the potential infiltration of outdoor air pollutants into indoor environments should also be considered, particularly in densely populated urban areas such as Seoul and Incheon, where various environmental factors coexist [70,71]. Older adults may be especially vulnerable to the adverse health effects of such exposures [72]. Among the key biological mechanisms involved, neuroinflammation refers to an inflammatory response triggered by the disruption of central nervous system homeostasis due to external stimuli. This process involves microglial activation and the release of pro-inflammatory cytokines, which can impair brain structure [73,74]. Block and Kim [75] found that exposure to air pollutants induced neuroinflammation, ultimately leading to neuronal damage and brain atrophy. In addition, oxidative stress and disruption of the blood–brain barrier (BBB) may serve as important interconnected mechanisms. Although these are distinct biological processes, they are closely linked in the context of air-pollution-induced neurotoxicity [76]. Oxidative stress has been shown to disrupt the integrity of the BBB by impairing endothelial tight junctions and increasing permeability, thereby allowing peripheral toxins to infiltrate the brain. These effects were demonstrated in both 2D and 3D in vitro BBB models [77], supporting a potential mechanism linking air pollution to neuroinflammation and structural brain changes.

This study had certain limitations. First, only individuals who participated in the EPINEP study were included; hence the small sample size and short research period limit the representativeness of the study in considering the entire population. To compensate for this limitation, we installed IoT equipment in each participant’s home to monitor and collect data on indoor air pollutants and to identify patterns in the activities of these individuals. We noted that the participants stayed indoors for more than 80% of the day and were also involved in various activities, such as household chores, social gatherings, workouts, and religious activities, similar to observations made in previous studies [78,79,80]. Nevertheless, the small sample size also limited the statistical power of the regression models, resulting in only a few statistically significant associations between indoor CO_2_ levels and selected brain MRI indicators. Second, given the cross-sectional nature of this study, only participants without neurological disorders were included, which limited the ability to capture changes over time. Therefore, future follow-up studies with an expanded scope will be necessary to examine long-term trends.

Third, although indoor exposure was estimated using IoT devices, the non-overlapping monitoring periods in the Incheon and Seoul cohorts limited between-cohort comparisons and seasonal adjustment. Hierarchical clustering was used to explore within-cohort heterogeneity, but this was not a substitute for seasonal confounding control. However, both cohorts had measurement durations exceeding three months, pollutant concentrations remained within regulatory limits, and brain structural measures were not expected to be highly sensitive to short-term fluctuations, so these intervals were considered reasonably stable exposure estimates. Future studies should aim for overlapping measurement periods and incorporate explicit seasonal adjustment and sensitivity analyses.

Given that the primary aim of this study was to analyze the correlation between indoor air pollutants and brain structure before and after brain volume correction, this difference likely had a negligible effect on the results obtained. Fourth, in this study, we analyzed the association between indoor air pollutants and brain-related indicators while making brain volume corrections. Thus, the results before and after correction showed similar trends, with no statistically significant differences detected in our analyses.

Some studies have indicated that variables reflecting head size, such as eTIV, may influence research outcomes, but evidence in this regard is still lacking [27,60,65,81,82]. Therefore, further research with expanded research targets is necessary.

Taken together, the findings of this study indicate that indoor air pollutants markedly affect brain structure (surface area, cortex thickness, and volume), and environmental factors are therefore associated with neurological disorders. Considering that environmental factors are modifiable, follow-up studies supplementing the limitations of this study may yield valuable results.

Given that older adults spend most of their time indoors and are physiologically more vulnerable to environmental stressors [83,84], the findings of this study highlight the urgent need to establish IAQ standards specifically tailored to elderly residential settings. Although many countries have implemented IAQ management guidelines for elderly-concentrated facilities such as nursing homes, regulatory frameworks for general residential environments remain limited [85,86,87]. Therefore, establishing regular IAQ monitoring systems in real-life residential spaces and implementing continuous education programs to raise awareness of indoor air quality are necessary [88,89]. These efforts could help preserve cognitive health in older adults and promote health equity from an environmental public health perspective.

## 5. Conclusions

In conclusion, our findings suggest that exposure to indoor air pollutants, including PM_2.5_, PM_10_, and CO_2_, may contribute to structural alterations in the brain regions involved in cognition and emotional regulation among older adults. These results highlight the need to develop targeted IAQ standards and implement continuous monitoring systems in elderly residential environments. Future longitudinal studies are warranted to validate these associations and to support public health strategies aimed at mitigating environmental risks to brain health.

## Figures and Tables

**Table 1:** Characteristics of study participants according to study design.

Variables	Total (Seoul + Incheon)	Seoul	Incheon
	N = 23	N = 14	N = 9
Age, mean ± SD (years)			
<75	11 (47.8)	8 (57.1)	3 (33.3)
≥75	12 (52.2)	6 (42.9)	6 (66.7)
Height, mean ± SD (cm)	156.2 (7.7)	159.5 (7.6)	151.2 (4.8)
Weight, mean ± SD (kg)	61.2 (7.4)	62.9 (7.7)	58.6 (6.4)
Gender, N (%)			
Male	7 (30.4)	7 (50)	0 (0)
Female	16 (69.6)	7 (50)	9 (100)
Education, N (%)			
High school graduate or below	5 (21.7)	3 (21.4)	2 (22.2)
High school graduate or above	18 (78.3)	11 (78.6)	7 (77.8)
Body mass index (BMI), mean ± SD (kg/m^2^)			
<25	13 (56.5)	8 (57.1)	5 (55.6)
≥25	10 (43.5)	6 (42.9)	4 (44.4)
Angina pectoris, N (%)			
Without diabetes	19 (82.6)	11 (78.6)	8 (88.9)
With diabetes	4 (17.4)	3 (21.4)	1 (11.1)
Hypertension, N (%)			
Without diabetes	6 (26.1)	4 (28.6)	2 (22.2)
With diabetes	17 (73.9)	10 (71.4)	7 (77.8)
Hyperlipidemia, N (%)			
Without diabetes	9 (39.1)	3 (21.4)	6 (66.7)
With diabetes	14 (60.9)	11 (78.6)	3 (33.3)
Diabetes, N (%)			
Without diabetes	18 (78.3)	9 (64.3)	9 (100)
With diabetes	5 (21.7)	5 (35.7)	0 (0.00)

SD, standard deviation. Values for continuous variables are presented as mean (SD); values for categorical variables are presented as sample size and percentages. Data are presented as mean ± standard deviation or N (%). High school graduate or below includes high school graduates and those who dropped out before completing primary or secondary education. Age categories were defined using a 75-year cutoff for subgroup analysis purposes. All participants were aged 65 years or older at enrollment.

**Table 2:** Summary statistics of new environmental variables.

Variable	Mean	Min	Max	Median	IQR
PM_2.5_ (µg/m^3^)	17.99	1.00	196.80	13.48	13.52
PM_10_ (µg/m^3^)	24.07	1.07	324.15	18.88	18.40
CO_2_ (ppm)	791.59	295.29	2554.54	688.44	427.42

**Table 3:** Association between indoor air pollutants and brain-related indicators before and after correction for cerebral volume (Models 1 and 2).

Variables		Model l						Model 2					
		Hippocampal Volume by Hemisphere											
		Left			Right			Left			Right		
		Estimate	95%CI	*p*-Value	Estimate	95%CI	*p*-Value	Estimate	95%CI	*p*-Value	Estimate	95%CI	*p*-Value
CO_2_	min	−2.83	(−5.73, 0.06)	0.05	−3.29	(−5.55, −1.03)	**0.01**	**−2.29**	**(−4.85, 0.26)**	**0.07**	−2.88	(−4.90, −0.86)	**0.01**
	mean	−0.88	(−1.74, −0.02)	0.05	−0.86	(−1.62, −0.10)	**0.03**	−0.77	(−1.49, −0.04)	**0.04**	−0.77	(−1.43, −0.10)	**0.03**
	max	−0.32	(−0.76, 0.13)	0.15	−0.33	(−0.72, 0.05)	0.08	−0.25	(−0.64, 0.14)	0.19	−0.28	(−0.63, 0.07)	0.11
	gmean	−1.02	(−1.94, −0.10)	**0.03**	−0.99	(−1.80, −0.19)	**0.02**	−0.88	(−1.66, −0.11)	**0.03**	−0.88	(−1.59, −0.17)	**0.02**

Model 1 shows unadjusted results, while Model 2 presents results adjusted for cerebral volume based on Model 1; Bold values indicate statistical significance (*p* < 0.05).

**Table 4:** Association between indoor air pollutants and brain-related indicators before and after correction for cerebral volume (Models 3 and 4).

Variables		Model 3									Model 4								
		Hippocampal Volume by Amygdaloid						Hippocampal Volume by Hippocampus			Hippocampal Volume by Amygdaloid						Hippocampal Volume by Hippocampus		
		Left			Right			Right			Left			Right			Right		
		Estimate	95%CI	*p*-Value	Estimate	95%CI	*p*-Value	Estimate	95%CI	*p*-Value	Estimate	95%CI	*p*-Value	Estimate	95%CI	*p*-Value	Estimate	95%CI	*p*-Value
PM_2.5_	mean	−283.24	(−496.78, −69.71)	**0.01**	−292.37	(−549.47, −35.27)	**0.03**	−544.55	(−1088.22, −0.88)	0.05	−272.09	(−449.34, −94.85)	**0.01**	−276.69	(−463.52, −89.86)	**0.01**	−516.53	(−971.50, −61.56)	**0.03**
	gmean	−79.35	(−345.08, 186.39)	0.52	−67.80	(−371.21, 235.60)	0.63	−21.78	(−643.27, 599.71)	0.94	−57.85	(−304.20, 188.50)	0.61	−37.67	(−294.54, 219.20)	0.75	33.36	(−523.34, 590.07)	0.90

Model 3 shows unadjusted results, while Model 4 presents results adjusted for cerebral volume based on Model 3; Bold values indicate statistical significance (*p* < 0.05).

## Data Availability

The data are not publicly available due to ethical and privacy restrictions, but are available from the corresponding author upon reasonable request.

## Supplementary Materials

The following supporting information can be downloaded at: https://www.mdpi.com/article/10.3390/brainsci15080868/s1, Table S1. Comprehensive Summary Statistics of New Environmental Variables; Table S2. Comprehensive Summary statistics of magnetic resonance imaging variables; Table S3. Association between indoor air pollutants and brain-related indicators without adjustment for cerebral volume (Model 1); Table S4. Association between indoor air pollutants and brain-related indicators after correction for cerebral volume (Model 2); Table S5. Association between indoor air pollutants and brain-related indicators without adjustment for cerebral volume (Model 3); Table S6. Association between indoor air pollutants and brain-related indicators after correction for cerebral volume (Model 4); Table S7. Summary Statistics of Magnetic Resonance Imaging Variables Using Hierarchical Clustering Groups; Table S8. Summary Statistics of Indoor Air Pollutants Using Hierarchical Clustering Groups.

## Abbreviations

The following abbreviations are used in this manuscript:

BMI	Body Mass Index
BBB	Blood–Brain Barrier
CO_2_	Carbon Dioxide
EPINEF	Environmental Pollution-Induced Neurological Effects
IAQ	Indoor Air Quality
IoT	Internet of Things
MRI	Magnetic Resonance Imaging
PM_10_	Particulate Matter With Aerodynamic Diameter ≤ 10 µm
PM_2.5_	Fine Particulate Matter With Aerodynamic Diameter ≤ 2.5 µm
VOCs	Volatile Organic Compounds
WHO	World Health Organization

## References

[1] IQAir (2024). World Air Quality Report. https://www.iqair.com/world-air-quality-report.

[2] World Health Organization (WHO) (2021). WHO Global Air Quality Guidelines: Particulate Matter (PM2.5 and PM10), Ozone, Nitrogen Dioxide, Sulfur Dioxide, and Carbon Monoxide. https://www.who.int/publications/i/item/9789240034228.

[3] World Health Organization (WHO) (2021). WHO Guidelines for Indoor Air Quality: Selected Pollutants. https://www.who.int/publications/i/item/9789289002134.

[4] Yang J., Nam I., Yun H., Kim J., Oh H.J., Lee D., Jeon S.M., Yoo S.H., Sohn J.R. (2015). Characteristics of indoor air quality at urban elementary schools in Seoul, Korea: Assessment of effect of surrounding environments. Atmos. Pollut. Res..

[5] Putri A.N.A.R., Salam R.A., Rachmawati L.M., Ramadhan A., Adiwidya A.S., Jalasena A., Chandra I. (2022). Spatial modelling of indoor air pollution distribution at home. J. Phys. Conf. Ser..

[6] United States Environmental Protection Agency (2021). Indoor Air Quality. https://www.epa.gov/report-environment/indoor-air-quality.

[7] Yasmin A., Ahmed I., Haider M., Md Hossain K., Motalib M.A., Md Hossain S. (2024). Characterizing indoor air quality and identifying factors influencing air quality at home microenvironment in Dhaka city. Indoor Environ..

[8] Rosario Filho N.A., Urrutia-Pereira M., D’Amato G., Cecchi L., Ansotegui I.J., Galán C., Pomés A., Murrieta-Aguttes M., Caraballo L., Rouadi P. (2021). Air pollution and indoor settings. World Allergy Organ. J..

[9] United States Environmental Protection Agency (1988). The Inside Story: A Guide to Indoor Air Quality. https://www.epa.gov/indoor-air-quality-iaq/inside-story-guide-indoor-air-quality.

[10] Calderón-Garcidueñas L., Ayala A. (2022). Air pollution, ultrafine particles, and your brain: Are combustion nanoparticle emissions and engineered nanoparticles causing preventable fatal neurodegenerative diseases and common neuropsychiatric outcomes?. Environ. Sci. Technol..

[11] Cho J., Jang H., Park H., Noh Y., Sohn J., Koh S.B., Lee S.K., Kim S.Y., Kim C. (2023). Alzheimer’s disease-like cortical atrophy mediates the effect of air pollution on global cognitive function. Environ. Int..

[12] Liu F., Liu C., Liu Y., Wang J., Wang Y., Yan B. (2023). Neurotoxicity of the air-borne particles: From molecular events to human diseases. J. Hazard. Mater..

[13] Jung C.R., Lin Y.T., Hwang B.F. (2015). Ozone, particulate matter, and newly-diagnosed Alzheimer’s disease: A population-based cohort study in Taiwan. J. Alzheimers Dis..

[14] Wilker E.H., Preis S.R., Beiser A.S., Wolf P.A., Au R., Kloog I., Li W., Schwartz J., Koutrakis P., DeCarli C. (2015). Long-term exposure to fine particulate matter, residential proximity to major roads and measures of brain structure. Stroke.

[15] Li W., Lin G., Xiao Z., Zhang Y., Li B., Zhou Y., Ma Y., Chai E. (2022). A review of respirable fine particulate matter (PM_2.5_)-induced brain damage. Front. Mol. Neurosci..

[16] Luo Q., Yang J., Yang M., Wang Y., Liu Y., Liu J., Kalvakolanu D.V., Cong X., Zhang J., Gui B. (2025). Utilization of nanotechnology to surmount the blood–brain barrier in disorders of the central nervous system. Mater. Today Bio..

[17] Health Chosun Editorial Team (2012). Why Do Older Adults Stay Indoors?. https://m.health.chosun.com/svc/news_view.html?contid=2012092801498.

[18] Julie C. (2012). Elevated Indoor Carbon Dioxide Impairs Decision-Making Performance.

[19] Li A., Tan T., Tang Y., Yang J., Cui D., Wang R., Wang A., Fei X., Di Y., Wang X. (2019). Endogenous formaldehyde is a memory-related molecule in mice and humans. Commun. Biol..

[20] Sarsilmaz M., Kaplan S., Songur A., Coş̧akoğlu S., Aslan H., Tunc A.T., Ozen O.A., Turgut M., Baş O. (2007). Effects of postnatal formaldehyde exposure on pyramidal cell number, volume of cell layer in hippocampus and hemisphere in the rat: A stereological study. Brain Res..

[21] Zheng J., Pang Y., Zhang Y., Hu W., Yang P., Liu Q., Ning J., Du Z., Jin X., Tang J. (2022). Indoor VOCs exposure induced Parkinson-like behaviors through autophagy dysfunction and NLRP3 inflammasome-mediated neuroinflammation. J. Hazard. Mater..

[22] Khan A., Kanwal H., Bibi S., Mushtaq S., Khan A., Khan Y.H., Mallhi H., Akash M.S.H., Rehman K. (2021). Volatile organic compounds and neurological disorders: From exposure to preventive interventions. Environmental Contaminants and Neurological Disorders.

[23] Chen Y., Cui P.Y., Pan Y.Y., Li Y.X., Waili N., Li Y. (2021). Association between housing environment and depressive symptoms among older people: A multidimensional assessment. BMC Geriatr..

[24] Obodunrin O.P. (2022). Location-based approach to determining the effective dose from radon concentrations in residential environments. J. Appl. Sci. Environ. Manag..

[25] National Institute of Environmental Research (2019). Korean Exposure Factor Handbook. https://ecolibrary.me.go.kr/nier/#/search/detail/5686028.

[26] Wang J., Hill-Jarrett T., Buto P., Pederson A., Sims K.D., Zimmerman S.C., DeVost M.A., Ferguson E., Lacar B., Yang Y. (2024). Comparison of approaches to control for intracranial volume in research on the association of brain volumes with cognitive outcomes. Hum. Brain Mapp..

[27] Hu M., Lou Y., Zhu C., Chen J., Liu S., Liang Y., Liu S., Tang Y. (2024). Evaluating the Impact of Intracranial Volume Correction Approaches on the Quantification of Intracranial Structures in MRI: A Systematic Analysis. J. Magn. Reson. Imaging.

[28] Kijonka M., Borys D., Psiuk-Maksymowicz K., Gorczewski K., Wojcieszek P., Kossowski B., Marchewka A., Swierniak A., Sokol M., Bobek-Billewicz B. (2020). Whole Brain and Cranial Size Adjustments in Volumetric Brain Analyses of Sex- and Age-Related Trends. Front. Neurosci..

[29] Canning T., la Torre J.A.-D., Fisher H.L., Gulliver J., Hansell A.L., Hardy R., Hatch S.L., Mudway I.S., Ronaldson A., Cartlidge M. (2025). Associations between life course exposure to ambient air pollution with cognition and later-life brain structure: A population-based study of the 1946 British Birth Cohort. Am. J. Med Sci..

[30] Thompson R., Tong X., Shen X., Ran J., Sun S., Yao X.I., Shen C. (2025). Longitudinal associations between air pollution and incident dementia as mediated by MRI-measured brain volumes in the UK Biobank. Environ. Int..

[31] Buckner R.L., Head D., Parker J., Fotenos A.F., Marcus D., Morris J.C., Snyder A.Z. (2009). A unified approach for morphometric and functional data analysis in young, old, and demented adults using automated atlas-based head size normalization: Reliability and validation against manual measurement of total intracranial volume. NeuroImage.

[32] Cho J., Sohn J., Noh J., Jang H., Kim W., Cho S.K., Seo H., Seo G., Lee S.K., Noh Y. (2020). Association between exposure to polycyclic aromatic hydrocarbons and brain cortical thinning: The Environmental Pollution-Induced Neurological EFfects (EPINEF) study. Sci. Total Environ..

[33] Cho J., Noh Y., Kim S.Y., Sohn J., Noh J., Kim W., Cho S.K., Seo H., Seo G., Lee S.K. (2020). Long-term ambient air pollution exposures and brain imaging markers in Korean adults: The Environmental Pollution-Induced Neurological Effects (EPINEF) study. Environ. Health Perspect..

[34] Lee T.J., Kim D.Y., Lee S.M., Kim S.C., Jo Y.M. (2021). Analysis of Indoor Air Quality Characteristics of Multi-use Facilities in Gyeonggi-do Using IoT-based Monitoring Data. J. Korean Soc. Atmos. Environ..

[35] Kim H.H. (2022). Exposure Characteristics of Indoor Air Pollutants in Some Local Public Buses. J. Environ. Health Sci..

[36] Ministry of Environment, Republic of Korea (2023). Official Test Method for Indoor Air Quality.

[37] National Institute of Environmental Research (2023). Performance Certification of Simplified Measurement Devices.

[38] Tang R., Sahu R., Su Y., Milsom A., Mishra A., Berkemeier T., Pfrang C. (2024). Impact of Cooking Methods on Indoor Air Quality: A Comparative Study of Particulate Matter (PM) and Volatile Organic Compound (VOC) Emissions. Indoor Air.

[39] Tormey D., Huntley S. (2023). The Effects of Cooking on Residential Indoor Air Quality: A Critical Review of the Literature with an Emphasis on the Use of Natural Gas Appliances. Catalyst Environmental Solutions Report.

[40] Alhajeri N.S., Alrashidi A.M., Yassin M.F., Al-Awadi L. (2024). Optimizing Indoor Air Quality: Investigating Particulate Matter Exposure in Household Kitchens and Source Identification. Environ. Qual. Manag..

[41] Crouse D.L., Peters P.A., van Donkelaar A., Goldberg M.S., Villeneuve P.J., Brion O., Khan S., Atari D.O., Jerrett M., Pope C.A. (2012). Risk of nonaccidental and cardiovascular mortality in relation to long-term exposure to low concentrations of fine particulate matter: A Canadian national-level cohort study. Environ. Health Perspect..

[42] Ha K.H., Suh M., Kang D.R., Kim H.C., Shin D.C., Kim C. (2011). Ambient particulate matter and the risk of deaths from cardiovascular and cerebrovascular disease. J. Korean Soc. Hypertens..

[43] Liao N.S., Sidney S., Deosaransingh K., van den Eeden S.K., Schwartz J., Alexeeff S.E. (2021). Particulate air pollution and risk of cardiovascular events among adults with a history of stroke or acute myocardial infarction. J. Am. Heart Assoc..

[44] Weichenthal S., Villeneuve P.J., Burnett R.T., Van Donkelaar A., Martin R.V., Jones R.R., DellaValle C.T., Sandler D.P., Ward M.H., Hoppin J.A. (2014). Long-term exposure to fine particulate matter: Association with nonaccidental and cardiovascular mortality in the agricultural health study cohort. Environ. Health Perspect..

[45] Ruan Y. (2024). Exploring multiple regression models: Key concepts and applications. Sci. Technol. Eng. Chem. Environ. Prot..

[46] R Core Team (2020). R: A Language and Environment for Statistical Computing.

[47] Ministry Ministry of Environment, Republic of Korea Indoor Air Quality Control Act (Act No. 19663), Enacted 16 August 2023; Effective 17 February 2024. Statutes of the Republic of Korea. https://elaw.klri.re.kr.

[48] Mendell M.J., Chen W., Ranasinghe D.R., Castorina R., Kumagai K. (2024). Carbon dioxide guidelines for indoor air quality: A review. J. Expo. Sci. Environ. Epidemiol..

[49] Balboni E., Filippini T., Crous-Bou M., Guxens M., Erickson L.D., Vinceti M. (2022). The association between air pollutants and hippocampal volume from magnetic resonance imaging: A systematic review and meta-analysis. Environ. Res..

[50] National Institute of Neurological Disorders and Stroke (2024). Dementias. https://www.ninds.nih.gov/health-information/disorders/dementias.

[51] Schuff N., Woerner N., Boreta L., Kornfield T., Shaw L.M., Trojanowski J.Q., Thompson P.M., Jack C.R., Weiner M.W. (2009). MRI of hippocampal volume loss in early Alzheimer’s disease in relation to ApoE genotype and biomarkers. Brain.

[52] Rostrup E., Law I., Blinkenberg M., Larsson H., Born A., Holm S., Paulson O. (2000). Regional Differences in the CBF and BOLD Responses to Hypercapnia: A Combined PET and fMRI Study. NeuroImage.

[53] Sicard K.M., Duong T.Q. (2005). Effects of hypoxia, hyperoxia, and hypercapnia on baseline and stimulus-evoked BOLD, CBF, and CMRO2 in spontaneously breathing animals. NeuroImage.

[54] Xu F., Uh J., Brier M.R., Hart J., Yezhuvath U.S., Gu H., Yang Y., Lu H. (2011). The influence of carbon dioxide on brain activity and metabolism in conscious humans. J. Cereb. Blood Flow Metab..

[55] Ziemann A.E., Allen J.E., Dahdaleh N.S., Drebot I.I., Coryell M.W., Wunsch A.M., Lynch C.M., Faraci F.M., Howard M.A., Welsh M.J. (2009). The Amygdala Is a Chemosensor that Detects Carbon Dioxide and Acidosis to Elicit Fear Behavior. Cell.

[56] Kiray M., Sisman A., Camsari U., Evren M., Dayi A., Baykara B., Aksu I., Ates M., Uysal N. (2013). Effects of carbon dioxide exposure on early brain development in rats. Biotech. Histochem..

[57] Fan Y., Cao X., Zhang J., Lai D., Pang L. (2023). Short-term exposure to indoor carbon dioxide and cognitive task performance: A systematic review and meta-analysis. Build. Environ..

[58] Ives J. (2020). Atmospheric CO2 Levels Can Cause Cognitive Impairment. News-Medical. https://www.news-medical.net/news/20200421/Atmospheric-CO2-levels-can-cause-cognitive-impairment.aspx.

[59] Allen J.G., MacNaughton P., Satish U., Santanam S., Vallarino J., Spengler J.D. (2016). Associations of cognitive function scores with carbon dioxide, ventilation, and volatile organic compound exposures in office workers: A controlled exposure study of green and conventional office environments. Environ. Health Perspect..

[60] Barnes J., Ridgway G.R., Bartlett J., Henley S.M., Lehmann M., Hobbs N., Clarkson M.J., MacManus D.G., Ourselin S., Fox N.C. (2010). Head size, age and gender adjustment in MRI studies: A necessary nuisance?. NeuroImage.

[61] Im K., Lee J.M., Lyttelton O., Kim S.H., Evans A.C., Kim S.I. (2008). Brain size and cortical structure in the adult human brain. Cereb. Cortex..

[62] Park Y., Kim S. (2015). The effects of age, gender, and head size on the cortical thickness of brain. Korean J. Biol. Psychiatry.

[63] Tae W.S., Lee E.K., Joo E.Y., Seo D.W., Hong S.B. (2003). Volume changes of frontal lobe and hippocampus in juvenile myoclonic epilepsy. J. Korean Neurol. Assoc..

[64] Im S., Lee J., Kim S., Shin C.-J., Son J.-W., Ju G., Lee S.-I. (2016). Surface-Based Parameters of Brain Imaging in Male Patients with Alcohol Use Disorder. Psychiatry Investig..

[65] Joo E.Y., Lee E.K., Tae W.S., Hong S.B. (2007). Hippocampal Volumetry in Normal Volunteers. Ewha Med J..

[66] Hong S.-J., Song J.-A., Jung S.-J., Jung Y.-J., Kim E.-Y., Noh H.-J., Joo Y.-S. (2009). A comparison and application of the clustering methods. Ann. Stat. Res..

[67] Gellhorn E. (1953). On the physiological action of carbon dioxide on cortex and hypothalamus. Electroencephalogr. Clin. Neurophysiol..

[68] Shi L., Steenland K., Li H., Liu P., Zhang Y., Lyles R.H., Requia W.J., Ilango S.D., Chang H.H., Wingo T. (2021). A national cohort study (2000–2018) of long-term air pollution exposure and incident dementia in older adults in the United States. Nat. Commun..

[69] Kawada T. (2014). Air pollution and the risk of stroke by meta-analysis. Int. J. Cardiol..

[70] Jung S.-H., Baek S.-H., Park S.-Y., Lee C.-M., Lee J.-I. (2025). Regional differences in PM_2_._5_ chemical composition and inhalation risk assessment: A case study of Seoul, Incheon, and Wonju. Toxics.

[71] Jones A.P. (1999). Indoor air quality and health. Atmos. Environ..

[72] European Environment Agency (2024). Air Quality in Europe—2024 Report.

[73] DiSabato D.J., Quan N., Godbout J.P. (2016). Neuroinflammation: The devil is in the details. J. Neurochem..

[74] Lane M., Oyster E., Luo Y., Wang H. (2025). The Effects of Air Pollution on Neurological Diseases: A Narrative Review on Causes and Mechanisms. Toxics.

[75] Block M.L., Calderón-Garcidueñas L. (2009). Air pollution: Mechanisms of neuroinflammation and CNS disease. Trends Neurosci..

[76] Hahad O., Lelieveld J., Birklein F., Lieb K., Daiber A., Münzel T. (2020). Ambient air pollution increases the risk of cerebrovascular and neuropsychiatric disorders through induction of inflammation and oxidative stress. Int. J. Mol. Sci..

[77] Chung T.D., Linville R.M., Guo Z., Ye R., Jha R., Grifno G.N., Searson P.C. (2022). Effects of acute and chronic oxidative stress on the blood–brain barrier in 2D and 3D in vitro models. Fluids Barriers CNS.

[78] Han H.-E., Park J.-H., Park J.-R., Lee D.-P. (2021). A Study on the Status and Satisfaction of the Elderly’s Participation in Leisure Activities. J. Green Ind. Res..

[79] National Institute of Environmental Research (2014). A Pilot Study on the Time-Activity Pattern for Exposure Pathway of Hazardous Pollutants (II). https://scienceon.kisti.re.kr/srch/selectPORSrchReport.do?cn=TRKO201500013892.

[80] Statistics Korea 2019 Time Use Survey. https://kostat.go.kr/board.es?mid=a20111060000&bid=11762&act=view&list_no=385431.

[81] Klasson N., Olsson E., Eckerström C., Malmgren H., Wallin A. (2018). Estimated intracranial volume from FreeSurfer is biased by total brain volume. Eur. Radiol. Exp..

[82] Sanchis-Segura C., Ibañez-Gual M.V., Aguirre N., Cruz-Gómez Á.J., Forn C. (2020). Effects of different intracranial volume correction methods on univariate sex differences in grey matter volume and multivariate sex prediction. Sci. Rep..

[83] World Health Organization (2010). Environment and Health Risks: A Review of the Influence and Effects of Social Inequalities.

[84] Spengler J.D., Sexton K. (1983). Indoor air pollution: A public health perspective. Science.

[85] Mata T.M., Felgueiras F., Martins A.A., Monteiro H., Ferraz M.P., Oliveira G.M., Gabriel M.F., Silva G.V. (2022). Indoor air quality in elderly centers: Pollutants emission and health effects. Environments.

[86] Reddy M., Heidarinejad M., Stephens B., Rubinstein I. (2021). Adequate indoor air quality in nursing homes: An unmet medical need. Sci. Total Environ..

[87] Rodríguez M., Seseña S., Valiente N., Palop M.L., Rodríguez A. (2024). Indoor air quality in elderly care centers: A multidisciplinary approach. Build. Environ..

[88] (2023). A review of critical residential buildings parameters and activities when investigating indoor air quality and pollutants. Build. Environ..

[89] Karottki D.G., Spilak M., Frederiksen M., Gunnarsen L., Brauner E.V., Kolarik B., Andersen Z.J., Sigsgaard T., Barregard L., Strandberg B. (2012). An indoor air filtration study in homes of elderly: Cardiovascular and respiratory effects of exposure to particulate matter. Environ. Health.

