# Supplementary material for: The Association Between Indoor Air Pollutants and Brain Structure Indicators Using eTIV-Adjusted and Unadjusted Models: A Study in Seoul and Incheon
Source: Brain Sci. 2025 Aug 14;15(8):868. doi: 10.3390/brainsci15080868 (PMC12384879; doi:10.3390/brainsci15080868)
Supplement: Supplementary file 1 [file brainsci-15-00868-s001.zip › brainsci-3786516-supplementary.pdf]

Association Between Indoor Air Pollutants and Brain Structure Indicators Identified Using Intracranial Volume-Adjusted and Unadjusted Models

Sun-Min An[a], Ho-Hyun Kim[a,b*]

Supplementary Tables

Supplementary Table S1. Comprehensive Summary Statistics of New Environmental Variables

Supplementary Table S2. Comprehensive Summary statistics of magnetic resonance imaging variables

Supplementary Table S3. Association between indoor air pollutants and brain-related indicators without adjustment for cerebral volume (Model 1)

Supplementary Table S4. Association between indoor air pollutants and brain-related indicators after correction for cerebral volume (Model 2)

Supplementary Table S5. Association between indoor air pollutants and brain-related indicators without adjustment for cerebral volume (Model 3)

Supplementary Table S6. Association between indoor air pollutants and brain-related indicators after correction for cerebral volume (Model 4)

Supplementary Table S7. Summary Statistics of Magnetic Resonance Imaging Variables Using Hierarchical Clustering Groups

Supplementary Table S8. Summary Statistics of Indoor Air Pollutants Using Hierarchical Clustering Groups

Supplementary Table S1. Comprehensive Summary Statistics of New Environmental Variables

Category		Total (Seoul+Incheon)		Seoul		Incheon	
		Mean (Min–Max)	Median (Q1–Q3)	Mean (Min–Max)	Median (Q1–Q3)	Mean (Min–Max)	Median (Q1–Q3)
$PM_{2.5}$ (μg/㎥)	min	2.8 (1.0–6.2)	2.6 (1.5–3.4)	3.2 (1.0–6.2)	3.1 (2.1–4.6)	2.1 (1.0–3.4)	2.1 (1.4–2.8)
	mean	17.8 (5.4–52.8)	14.7 (13.2–19.9)	17.2 (5.4–27.5)	16.2 (13.8–20.3)	18.7 (9.7–52.8)	14.3 (12.9–15.4)
	max	76.6 (19.2–196.8)	60.9 (44.6–105.3)	73.6 (19.2–196.8)	62.3 (38.9–81.5)	81.1 (44.5–153.5)	57.7 (51.8–125.8)
	gm	14.2 (4.3–35.9)	12.6 (10.7–16.5)	14.1 (4.3–21.8)	13.8 (12.1–16.6)	14.4 (7.1–35.9)	11.9 (10.2–12.7)
PM_{10} (μg/㎥)	min	3.7 (1.1–8.1)	3.4 (2.1–4.8)	4.5 (1.1–8.1)	4.4 (2.4–5.8)	2.6 (1.4–4.1)	2.3 (2.0–3.4)
	mean	23.9 (8.1–61.7)	22.4 (18.9–26.8)	23.8 (8.1–35.5)	22.8 (20.3–27.2)	24.2 (11.7–61.7)	18.7 (15.8–22.9)
	max	121.4 (28.5–324.2)	77.7 (59.7–165.1)	115.1 (28.5–264.8)	92.9 (54.8–165.1)	131.2 (62.6–324.2)	76.2 (68.4–124.9)
	gm	19.4 (6.4–41.6)	19.3 (15.6–22.1)	19.8 (6.4–32.2)	19.7 (17.3–22.2)	18.7 (8.4–41.6)	15.9 (12.5–18.9)
CO_2 (ppm)	min	456.2 (295.3–628.0)	438.1 (412.4–481.8)	456.9 (295.3–628.0)	433.4 (411.4–513.8)	455.0 (363.4–605.6)	450.3 (430.0–462.4)
	mean	772.2 (431.7–1597.9)	743.4 (591.3–881.1)	692.4 (431.7–1090.7)	655.8 (584.5–779.8)	896.5 (528.7–1597.9)	878.6 (814.6–885.3)
	max	1369.9 (551.8–2554.5)	1367.5 (957.2–1546.7)	1259.9 (551.8–2554.5)	1323.7 (897.9–1421.7)	1541.0 (768.4–2455.0)	1525.1 (1323.3–1840.4)
	gm	740.8 (430.5–1511.2)	714.7 (577.0–821.7)	669.2 (430.5–1062.0)	629.7 (563.3–765.0)	852.0 (522.8–1511.2)	809.1 (790.9–860.9)

Data measured in 5-min intervals were restructured into 24-h (1-day) intervals for analysis

Supplementary Table S2. Comprehensive Summary statistics of magnetic resonance imaging variables

Category		Total (Seoul+Incheon)		Seoul		Incheon	
		Mean (Min–Max)	Median (Q1–Q3)	Mean (Min–Max)	Median (Q1–Q3)	Mean (Min–Max)	Median (Q1–Q3)
Cerebral volume size (mm^3)		1,530,571 (1,100,356–1,951,157)	1,526,651 (1,424,833–1,635,993)	1,549,593 (1,317,459–1,951,157)	1,545,131 (1,438,687–1,621,220)	1,449,883 (1,100,356–1,735,185)	1,429,593 (1,393,001–1,526,651)
Cerebral surface area (mm^2)	Left hemisphere	75,784 (57,261–94,872)	75,376 (69,572–81,376)	77,369 (63,024–94,872)	77,919 (73,095–81,399)	71,032 (57,261–82,876)	73,206 (66,430–74,972)
	Right hemisphere	75,876 (58,119–95,960)	76,527 (69,758–81,827)	77,735 (61,997–95,960)	77,137 (74,261–81,847)	71,428 (58,119–83,190)	71,998 (67,018–75,304)
Cerebral cortex thickness (mm)	Overall average	2.35 (2.20–2.53)	2.33 (2.29–2.42)	2.34 (2.20–2.48)	2.33 (2.28–2.36)	2.38 (2.20–2.50)	2.39 (2.32–2.44)
	Left hemisphere average	2.35 (2.19–2.53)	2.34 (2.29–2.43)	2.34 (2.19–2.48)	2.33 (2.29–2.41)	2.38(2.20–2.48)	2.41 (2.32–2.46)
	Right hemisphere average	2.35 (2.21–2.54)	2.33 (2.29–2.42)	2.33 (2.22–2.48)	2.32 (2.29–2.36)	2.37 (2.21–2.52)	2.37 (2.31–2.44)
	Left hemisphere frontal lobe	2.50 (2.30–2.69)	2.47 (2.43–2.60)	2.47 (2.32–2.61)	2.45 (2.42–2.53)	2.55 (2.36–2.67)	2.59 (2.46–2.63)
	Right hemisphere frontal lobe	2.47 (2.27–2.71)	2.46 (2.40–2.54)	2.44 (2.27–2.58)	2.44 (2.40–2.50)	2.51 (2.33–2.71)	2.53 (2.46–2.56)
	Left hemisphere parietal lobe	2.16 (1.97–2.36)	2.18 (2.07–2.23)	2.14 (1.97–2.31)	2.15 (2.06–2.22)	2.19 (2.03–2.29)	2.21 (2.19–2.24)
	Right hemisphere parietal lobe	2.15 (1.99–2.33)	2.12 (2.09–2.20)	2.14 (2.03–2.29)	2.12 (2.08–2.20)	2.17 (2.02–2.29)	2.20 (2.12–2.20)
	Left hemisphere temporal lobe	2.68 (2.50–2.93)	2.68 (2.60–2.79)	2.66 (2.50–2.93)	2.68 (2.59–2.70)	2.72 (2.54–2.84)	2.78 (2.65–2.80)
	Right hemisphere temporal lobe	2.69 (2.50–2.92)	2.66 (2.63–2.74)	2.67 (2.50–2.92)	2.66 (2.64–2.70)	2.72 (2.54–2.89)	2.69 (2.68–2.78)
	Left hemisphere occipital lobe	1.79 (1.64–1.95)	1.79 (1.73–1.86)	1.80 (1.71–1.95)	1.80 (1.73–1.87)	1.78 (1.64–1.91)	1.79 (1.73–1.83)
	Right hemisphere occipital lobe	1.84 (1.74–2.00)	1.83 (1.78–1.90)	1.84 (1.74–1.99)	1.82 (1.77–1.89)	1.84 (1.75–1.95)	1.84 (1.76–1.90)
	Left hemisphere cingulate gyrus	2.43 (2.02–2.80)	2.40 (2.35–2.51)	2.43 (2.23–2.80)	2.40 (2.35–2.48)	2.41 (2.02–2.69)	2.37 (2.29–2.55)
	Right hemisphere cingulate gyrus	2.43 (1.99–2.69)	2.42 (2.32–2.54)	2.41 (2.24–2.68)	2.39 (2.33–2.50)	2.37 (1.99–2.66)	2.38 (2.27–2.52)
	Left hemisphere insular lobe	2.83 (2.54–3.13)	2.81 (2.70–2.91)	2.83 (2.54–3.13)	2.81 (2.71–2.91)	2.79 (2.57–3.12)	2.76 (2.70–2.84)
	Right hemisphere insular lobe	2.86 (2.37–3.34)	2.85 (2.72–2.95)	2.82 (2.37–3.24)	2.87 (2.71–2.94)	2.92 (2.61–3.34)	2.85 (2.78–3.08)
Subcortical volume (mm^3)	Left hemisphere nucleus accumbens	299.8 (165.4–407.8)	301.1 (277.4–342.6)	323.80 (199–407.80)	322.5 (302.5–360)	257.8 (165.4–335.2)	279.1 (216.3–290.4)
	Right hemisphere nucleus accumbens	430.9 (260.6–717.4)	418.7 (392.5–454.2)	440 (331–570.50)	422.5 (410.9–458.3)	378 (260.6–506.3)	394.3 (323.4–415.9)
	Left hemisphere amygdaloid body	1,284 (978–1,590)	1,298 (1,143–1,400)	1,302 (1,086–1,590)	1,325 (1,142–1,401)	1,244 (978–1,524)	1,243 (1,096–1,365)
	Right hemisphere amygdaloid body	1,436 (1,107–1,902)	1,395 (1,293–1,576)	1,470 (1,183–1,902)	1,437 (1,316–1,576)	1,366 (1,107–1,646)	1,318 (1,275–1,566)
	Left hemisphere hippocampus	3,498 (2,639–4,276)	3,456 (3,279–3,767)	3,551 (2,639–4,276)	3,580 (3,356–3,752)	3,426 (2,680–3,981)	3,388 (3,263–3,776)
	Right hemisphere hippocampus	3,688 (2,980–4,461)	3,653 (3,439–4,066)	3,774 (2,980–4,461)	3,755 (3,542–4,112)	3,647 (3,052–4,081)	3,612 (3,466–3,879)
	Left hemisphere globus pallidus	1,835 (1,454–2,202)	1,830 (1,629–2,043)	1,892 (1,497–2,202)	1,881 (1,723–2,087)	1,748 (1,454–2,094)	1,660 (1,588–1,999)
	Right hemisphere globus pallidus	1,717 (1,372–2,059)	1,736 (1,534–1,855)	1,743 (1,410–2,059)	1,741 (1,636–1,854)	1,691 (1,372–2,032)	1,582 (1,515–1,900)
	Left hemisphere putamen	4,004 (3,196–4,951)	4,028 (3,714–4,328)	4,093 (3,492–4,951)	4,040 (3,767–4,306)	3,726 (3,196–4,374)	3,560 (3,324–4,190)
	Right hemisphere putamen	4,210 (3,364–6,051)	4,245 (3,775–4,447)	4,355 (3,742–6,051)	4,247 (3,985–4,562)	3,916 (3,364–4,742)	3,751 (3,682–4,258)
	Left hemisphere caudate nucleus	2,917 (2,404–3,612)	2,863 (2,613–3,223)	2,989 (2,543–3,612)	2,874 (2,732–3,245)	2,724 (2,404–3,277)	2,589 (2,440–2,954)
	Right hemisphere caudate nucleus	3,080 (2,489–3,743)	2,967 (2,751–3,405)	3,121 (2,489–3,743)	3,041 (2,816–3,419)	2,911 (2,498–3,511)	2,763 (2,644–3,199)
	Left hemisphere thalamus	6,348 (4,810–7,706)	6,388 (6,051–6,769)	6,486 (5,222–7,706)	6,290 (6,113–7,095)	5,999 (4,810–7,078)	6,075 (5,362–6,443)
	Right hemisphere thalamus	5,954 (4,687–7,549)	5,989 (5,429–6,238)	6,077 (5,202–7,549)	5,967 (5,544–6,257)	5,714 (4,687–6,448)	5,859 (5,330–6,109)

Supplementary Table S3. Association between indoor air pollutants and brain-related indicators without adjustment for cerebral volume (Model 1)

Variable		Cerebral surface area						Cerebral cortex thickness								
		Left hemisphere			Right hemisphere			Left hemisphere average			Right hemisphere average			Overall average		
		estimate	95%CI	p-value	estimate	95%CI	p-value	estimate	95%CI	p-value	estimate	95%CI	p-value	estimate	95%CI	p-value
$PM_{2.5}$	min	-728.55	(-5109.98, 3652.88)	0.72	-793.72	(-5141.77, 3554.33)	0.70	0.01	(-0.04, 0.05)	0.74	0.00	(-0.04, 0.05)	0.80	0.01	(-0.03, 0.05)	0.76
	mean	36.37	(-489.85, 562.59)	0.88	47.73	(-474.72, 570.18)	0.84	0.00	(-0.00, 0.01)	0.18	0.00	(-0.00, 0.01)	0.07	0.00	(-0.00, 0.01)	0.10
	max	-96.38	(-266.16, 73.39)	0.24	-85.45	(-256.56, 85.67)	0.30	0.00	(-0.00, 0.00)	0.24	0.00	(-0.00, 0.00)	0.29	0.00	(-0.00, 0.00)	0.25
	gm	101.62	(-731.58, 934.82)	0.79	119.23	(-707.57, 946.04)	0.76	0.00	(-0.00, 0.01)	0.25	0.01	(-0.00, 0.01)	0.06	0.01	(-0.00, 0.01)	0.12
PM_{10}	min	-663.79	(-3965.68, 2638.10)	0.67	-688.84	(-3966.97, 2589.28)	0.65	0.00	(-0.03, 0.03)	1.00	0.00	(-0.03, 0.03)	0.97	0.00	(-0.03, 0.03)	0.99
	mean	26.17	(-453.39, 505.73)	0.91	42.06	(-433.94, 518.05)	0.85	0.00	(-0.00, 0.01)	0.20	0.00	(-0.00, 0.01)	0.08	0.00	(-0.00, 0.01)	0.12
	max	-21.16	(-92.02, 49.69)	0.52	-19.21	(-89.84, 51.42)	0.56	0.00	(-0.00, 0.00)	0.13	0.00	(-0.00, 0.00)	0.06	0.00	(-0.00, 0.00)	0.08
	gm	81.38	(-689.90, 852.66)	0.82	110.16	(-654.58, 874.91)	0.76	0.00	(-0.00, 0.01)	0.29	0.01	(-0.00, 0.01)	0.09	0.00	(-0.00, 0.01)	0.16
CO_2	min	-21.44	(-78.43, 35.55)	0.43	-22.48	(-78.91, 33.94)	0.40	0.00	(-0.00, 0.00)	0.32	0.00	(-0.00, 0.00)	0.16	0.00	(-0.00, 0.00)	0.22
	mean	-5.59	(-22.92, 11.74)	0.49	-5.90	(-23.06, 11.27)	0.47	0.00	(-0.00, 0.00)	0.47	0.00	(-0.00, 0.00)	0.11	0.00	(-0.00, 0.00)	0.24
	max	-2.03	(-10.27, 6.22)	0.60	-2.22	(-10.39, 5.95)	0.56	0.00	(-0.00, 0.00)	0.37	0.00	(-0.00, 0.00)	0.09	0.00	(-0.00, 0.00)	0.19
	gm	-6.71	(-25.69, 12.26)	0.45	-7.07	(-25.86, 11.73)	0.43	0.00	(-0.00, 0.00)	0.49	0.00	(-0.00, 0.00)	0.12	0.00	(-0.00, 0.00)	0.25

Supplementary Table S3. Association between indoor air pollutants and brain-related indicators without adjustment for cerebral volume (Model 1)(Continued)

Variable		Cerebral cortex thickness														
		Left hemisphere frontal lobe			Right hemisphere frontal lobe			Left hemisphere parietal lobe			Right hemisphere parietal lobe			Left hemisphere temporal lobe		
		estimate	95%CI	p-value	estimate	95%CI	p-value	estimate	95%CI	p-value	estimate	95%CI	p-value	estimate	95%CI	p-value
$PM_{2.5}$	min	0.00	(-0.05, 0.05)	0.96	-0.01	(-0.06, 0.04)	0.67	0.00	(-0.05, 0.05)	0.97	0.01	(-0.03, 0.05)	0.54	0.04	(-0.01, 0.09)	0.15
	mean	0.01	(-0.00, 0.01)	0.05	0.01	(0.00, 0.01)	0.04	0.00	(-0.00, 0.01)	0.54	0.00	(-0.00, 0.01)	0.18	0.00	(-0.00, 0.01)	0.13
	max	0.00	(-0.00, 0.00)	0.07	0.00	(-0.00, 0.00)	0.11	0.00	(-0.00, 0.00)	0.59	0.00	(-0.00, 0.00)	0.43	0.00	(-0.00, 0.00)	0.20
	gm	0.01	(-0.00, 0.02)	0.10	0.01	(0.00, 0.02)	0.05	0.00	(-0.01, 0.01)	0.66	0.00	(-0.00, 0.01)	0.13	0.01	(-0.00, 0.02)	0.13
PM_{10}	min	0.00	(-0.04, 0.03)	0.79	-0.01	(-0.05, 0.03)	0.49	-0.01	(-0.04, 0.03)	0.65	0.00	(-0.03, 0.03)	0.78	0.02	(-0.02, 0.06)	0.22
	mean	0.00	(-0.00, 0.01)	0.05	0.00	(0.00, 0.01)	0.05	0.00	(-0.00, 0.01)	0.64	0.00	(-0.00, 0.01)	0.24	0.00	(-0.00, 0.01)	0.12
	max	0.00	(0.00, 0.00)	0.04	0.00	(0.00, 0.00)	0.04	0.00	(-0.00, 0.00)	0.44	0.00	(-0.00, 0.00)	0.10	0.00	(-0.00, 0.00)	0.06
	gm	0.01	(-0.00, 0.01)	0.11	0.01	(-0.00, 0.01)	0.07	0.00	(-0.01, 0.01)	0.83	0.00	(-0.00, 0.01)	0.22	0.01	(-0.00, 0.02)	0.12
CO_2	min	0.00	(-0.00, 0.00)	0.53	0.00	(-0.00, 0.00)	0.15	0.00	(-0.00, 0.00)	0.24	0.00	(-0.00, 0.00)	0.24	0.00	(-0.00, 0.00)	0.53
	mean	0.00	(-0.00, 0.00)	0.47	0.00	(0.00, 0.00)	0.04	0.00	(-0.00, 0.00)	0.54	0.00	(-0.00, 0.00)	0.22	0.00	(-0.00, 0.00)	0.51
	max	0.00	(-0.00, 0.00)	0.34	0.00	(0.00, 0.00)	0.03	0.00	(-0.00, 0.00)	0.30	0.00	(-0.00, 0.00)	0.16	0.00	(-0.00, 0.00)	0.53
	gm	0.00	(-0.00, 0.00)	0.52	0.00	(-0.00, 0.00)	0.05	0.00	(-0.00, 0.00)	0.56	0.00	(-0.00, 0.00)	0.23	0.00	(-0.00, 0.00)	0.51

Supplementary Table S3. Association between indoor air pollutants and brain-related indicators without adjustment for cerebral volume (Model 1)(Continued)

Variable		Cerebral cortex thickness														
		Right hemisphere temporal lobe			Left hemisphere occipital lobe			Right hemisphere occipital lobe			Left hemisphere cingulate gyrus			Right hemisphere cingulate gyrus		
		estimate	95%CI	p-value	estimate	95%CI	p-value	estimate	95%CI	p-value	estimate	95%CI	p-value	estimate	95%CI	p-value
$PM_{2.5}$	min	0.02	(-0.03, 0.07)	0.34	0.01	(-0.03, 0.04)	0.58	-0.01	(-0.04, 0.03)	0.68	-0.03	(-0.13, 0.07)	0.54	0.05	(-0.02, 0.12)	0.15
	mean	0.00	(-0.00, 0.01)	0.12	0.00	(-0.01, 0.00)	0.60	0.00	(-0.01, 0.00)	0.31	0.00	(-0.01, 0.01)	0.84	0.00	(-0.00, 0.01)	0.28
	max	0.00	(-0.00, 0.00)	0.48	0.00	(-0.00, 0.00)	0.61	0.00	(-0.00, 0.00)	0.11	0.00	(-0.01, 0.00)	0.60	0.00	(-0.00, 0.00)	0.53
	gm	0.01	(-0.00, 0.02)	0.11	0.00	(-0.01, 0.01)	0.61	0.00	(-0.01, 0.00)	0.29	0.00	(-0.02, 0.02)	0.96	0.01	(-0.01, 0.02)	0.30
PM_{10}	min	0.01	(-0.03, 0.05)	0.47	0.01	(-0.02, 0.03)	0.56	-0.01	(-0.03, 0.02)	0.65	-0.02	(-0.09, 0.05)	0.55	0.04	(-0.02, 0.09)	0.14
	mean	0.00	(-0.00, 0.01)	0.12	0.00	(-0.00, 0.00)	0.63	0.00	(-0.01, 0.00)	0.28	0.00	(-0.01, 0.01)	0.76	0.00	(-0.00, 0.01)	0.27
	max	0.00	(-0.00, 0.00)	0.13	0.00	(-0.00, 0.00)	0.43	0.00	(-0.00, 0.00)	0.24	0.00	(-0.00, 0.00)	0.64	0.00	(-0.00, 0.00)	0.27
	gm	0.01	(-0.00, 0.01)	0.12	0.00	(-0.01, 0.00)	0.68	0.00	(-0.01, 0.00)	0.27	0.00	(-0.02, 0.02)	0.87	0.01	(-0.01, 0.02)	0.27
CO_2	min	0.00	(-0.00, 0.00)	0.57	0.00	(-0.00, 0.00)	0.89	0.00	(-0.00, 0.00)	0.34	0.00	(-0.00, 0.00)	0.15	0.00	(-0.00, 0.00)	0.48
	mean	0.00	(-0.00, 0.00)	0.32	0.00	(-0.00, 0.00)	0.92	0.00	(-0.00, 0.00)	0.64	0.00	(-0.00, 0.00)	0.33	0.00	(-0.00, 0.00)	0.83
	max	0.00	(-0.00, 0.00)	0.33	0.00	(-0.00, 0.00)	0.65	0.00	(-0.00, 0.00)	0.69	0.00	(-0.00, 0.00)	0.42	0.00	(-0.00, 0.00)	0.51
	gm	0.00	(-0.00, 0.00)	0.34	0.00	(-0.00, 0.00)	1.00	0.00	(-0.00, 0.00)	0.60	0.00	(-0.00, 0.00)	0.33	0.00	(-0.00, 0.00)	0.87

Supplementary Table S3. Association between indoor air pollutants and brain-related indicators without adjustment for cerebral volume (Model 1)(Continued)

Variable		Cerebral cortex thickness						Subcortical volume								
		Left hemisphere insular lobe			Right hemisphere insular lobe			Left hemisphere nucleus accumbens			Right hemisphere nucleus accumbens			Left hemisphere amygdaloid body		
		estimate	95%CI	p-value	estimate	95%CI	p-value	estimate	95%CI	p-value	estimate	95%CI	p-value	estimate	95%CI	p-value
$PM_{2.5}$	min	-0.03	(-0.12, 0.07)	0.58	-0.02	(-0.17, 0.13)	0.81	30.86	(4.17, 57.56)	0.03	5.39	(-41.88, 52.67)	0.81	-58.28	(-153.53, 36.97)	0.21
	mean	0.01	(-0.00, 0.02)	0.15	0.01	(-0.01, 0.03)	0.26	0.90	(-3.07, 4.88)	0.63	2.40	(-3.04, 7.84)	0.35	2.74	(-9.41, 14.89)	0.63
	max	0.00	(-0.00, 0.00)	0.68	0.00	(-0.01, 0.01)	0.72	0.46	(-0.89, 1.81)	0.47	0.03	(-1.93, 1.98)	0.98	-0.34	(-4.56, 3.89)	0.86
	gm	0.01	(-0.01, 0.03)	0.34	0.02	(-0.01, 0.04)	0.16	1.42	(-4.89, 7.73)	0.63	3.96	(-4.64, 12.55)	0.33	2.17	(-17.27, 21.61)	0.81
PM_{10}	min	-0.03	(-0.10, 0.04)	0.35	-0.02	(-0.13, 0.09)	0.72	18.70	(-3.49, 40.89)	0.09	-0.96	(-36.78, 34.86)	0.95	-56.10	(-124.29, 12.09)	0.10
	mean	0.01	(-0.00, 0.02)	0.18	0.01	(-0.01, 0.02)	0.28	0.56	(-3.09, 4.20)	0.74	2.10	(-2.87, 7.07)	0.37	2.18	(-8.92, 13.28)	0.67
	max	0.00	(-0.00, 0.00)	0.23	0.00	(-0.00, 0.00)	0.19	0.31	(-0.20, 0.82)	0.21	0.24	(-0.52, 1.00)	0.50	-0.32	(-1.99, 1.35)	0.68
	gm	0.01	(-0.01, 0.02)	0.46	0.01	(-0.01, 0.04)	0.22	0.83	(-5.05, 6.70)	0.76	3.33	(-4.69, 11.34)	0.38	0.84	(-17.18, 18.87)	0.92
CO_2	min	0.00	(-0.00, 0.00)	0.72	0.00	(-0.00, 0.00)	0.32	0.12	(-0.32, 0.57)	0.55	0.03	(-0.60, 0.66)	0.93	-0.85	(-2.10, 0.40)	0.16
	mean	0.00	(-0.00, 0.00)	0.72	0.00	(-0.00, 0.00)	0.20	-0.02	(-0.15, 0.12)	0.76	-0.04	(-0.23, 0.15)	0.65	-0.19	(-0.59, 0.20)	0.30
	max	0.00	(-0.00, 0.00)	0.53	0.00	(-0.00, 0.00)	0.22	0.01	(-0.06, 0.07)	0.85	-0.01	(-0.10, 0.08)	0.80	-0.05	(-0.24, 0.14)	0.55
	gm	0.00	(-0.00, 0.00)	0.79	0.00	(-0.00, 0.00)	0.21	-0.02	(-0.17, 0.12)	0.74	-0.05	(-0.26, 0.16)	0.62	-0.23	(-0.66, 0.19)	0.25

Supplementary Table S3. Association between indoor air pollutants and brain-related indicators without adjustment for cerebral volume (Model 1)(Continued)

Variable		Subcortical volume														
		Right hemisphere amygdaloid body			Left hemisphere hippocampus			Right hemisphere hippocampus			Left hemisphere globus pallidus			Right hemisphere globus pallidus		
		estimate	95%CI	p-value	estimate	95%CI	p-value	estimate	95%CI	p-value	estimate	95%CI	p-value	estimate	95%CI	p-value
$PM_{2.5}$	min	-37.65	(-151.34, 76.03)	0.48	-56.23	(-312.57, 200.11)	0.64	-51.72	(-285.12, 181.69)	0.64	15.89	(-150.93, 182.72)	0.84	-10.47	(-155.10, 134.16)	0.88
	mean	1.62	(-12.24, 15.49)	0.80	-0.72	(-31.68, 30.23)	0.96	1.92	(-26.24, 30.09)	0.88	7.52	(-11.83, 26.86)	0.41	9.91	(-6.09, 25.92)	0.20
	max	-1.66	(-6.33, 3.00)	0.45	-3.13	(-13.59, 7.33)	0.52	-2.01	(-11.63, 7.61)	0.65	-1.40	(-8.21, 5.42)	0.66	0.84	(-5.10, 6.77)	0.76
	gm	1.56	(-20.49, 23.61)	0.88	-2.80	(-51.89, 46.29)	0.90	0.38	(-44.35, 45.12)	0.99	11.96	(-18.73, 42.65)	0.41	14.74	(-10.91, 40.40)	0.23
PM_{10}	min	-40.11	(-123.94, 43.73)	0.31	-64.62	(-255.62, 126.38)	0.47	-56.13	(-230.48, 118.21)	0.49	7.57	(-118.65, 133.79)	0.90	-18.57	(-127.30, 90.16)	0.71
	mean	1.02	(-11.63, 13.68)	0.86	-0.69	(-28.89, 27.50)	0.96	2.39	(-23.24, 28.03)	0.84	7.60	(-9.88, 25.08)	0.36	9.26	(-5.25, 23.78)	0.19
	max	-0.61	(-2.48, 1.25)	0.48	-1.08	(-5.27, 3.10)	0.58	-1.08	(-4.88, 2.72)	0.55	0.15	(-2.58, 2.89)	0.90	0.94	(-1.35, 3.23)	0.39
	gm	0.22	(-20.19, 20.64)	0.98	-3.16	(-48.55, 42.22)	0.88	1.31	(-40.06, 42.68)	0.95	13.06	(-14.94, 41.07)	0.33	13.98	(-9.66, 37.63)	0.22
CO_2	min	-1.02	(-2.42, 0.37)	0.14	-2.83	(-5.73, 0.06)	0.05	-3.29	(-5.55, -1.03)	**0.01**	-0.28	(-2.50, 1.94)	0.78	0.10	(-1.83, 2.03)	0.91
	mean	-0.24	(-0.68, 0.20)	0.25	-0.88	(-1.74, -0.02)	0.05	-0.86	(-1.62, -0.10)	**0.03**	-0.17	(-0.84, 0.49)	0.58	-0.06	(-0.64, 0.52)	0.83
	max	-0.08	(-0.30, 0.13)	0.40	-0.32	(-0.76, 0.13)	0.15	-0.33	(-0.72, 0.05)	0.08	-0.07	(-0.38, 0.24)	0.63	0.02	(-0.26, 0.29)	0.90
	gm	-0.28	(-0.76, 0.20)	0.22	-1.02	(-1.94, -0.10)	**0.03**	-0.99	(-1.80, -0.19)	**0.02**	-0.21	(-0.94, 0.51)	0.53	-0.09	(-0.73, 0.54)	0.75

Supplementary Table S3. Association between indoor air pollutants and brain-related indicators without adjustment for cerebral volume (Model 1)(Continued)

Variable		Subcortical volume																	
		Left hemisphere putamen			Right hemisphere putamen			Left hemisphere caudate nucleus			Right hemisphere caudate nucleus			Left hemisphere thalamus			Right hemisphere thalamus		
		estimate	95%CI	p-value	estimate	95%CI	p-value	estimate	95%CI	p-value	estimate	95%CI	p-value	estimate	95%CI	p-value	estimate	95%CI	p-value
$PM_{2.5}$	min	-54.46	(-313.69, 204.78)	0.65	36.86	(-344.38, 418.09)	0.84	34.42	(-192.27, 261.10)	0.74	-71.84	(-310.54, 166.86)	0.52	-18.93	(-453.14, 415.29)	0.93	-11.38	(-389.13, 366.38)	0.95
	mean	8.44	(-22.34, 39.21)	0.56	18.41	(-25.58, 62.39)	0.38	14.78	(-10.62, 40.18)	0.23	17.22	(-9.54, 43.97)	0.18	-12.54	(-63.78, 38.70)	0.60	-1.74	(-46.87, 43.40)	0.93
	max	-3.33	(-13.87, 7.22)	0.50	2.09	(-13.57, 17.76)	0.77	-1.40	(-10.73, 7.93)	0.75	-2.57	(-12.44, 7.30)	0.58	-8.67	(-25.60, 8.26)	0.28	-6.23	(-21.22, 8.76)	0.38
	gm	16.40	(-32.03, 64.83)	0.47	19.09	(-52.23, 90.42)	0.57	20.69	(-20.27, 61.66)	0.29	24.90	(-18.21, 68.00)	0.23	-22.26	(-103.30, 58.78)	0.56	0.41	(-71.24, 72.06)	0.99
PM_{10}	min	-39.89	(-235.91, 156.13)	0.66	2.46	(-286.23, 291.14)	0.99	21.70	(-149.87, 193.27)	0.79	-51.62	(-232.35, 129.11)	0.54	-51.23	(-377.74, 275.28)	0.74	-17.18	(-302.48, 268.11)	0.90
	mean	10.08	(-17.62, 37.78)	0.44	18.82	(-20.85, 58.49)	0.32	14.86	(-7.90, 37.62)	0.18	17.25	(-6.65, 41.15)	0.14	-12.73	(-59.26, 33.80)	0.56	-0.43	(-41.56, 40.70)	0.98
	max	0.29	(-4.00, 4.57)	0.89	1.55	(-4.62, 7.73)	0.59	-0.10	(-3.84, 3.63)	0.95	-0.26	(-4.25, 3.72)	0.89	-2.25	(-9.21, 4.71)	0.49	-2.19	(-8.21, 3.83)	0.44
	gm	20.38	(-23.49, 64.25)	0.33	22.88	(-42.38, 88.15)	0.46	22.79	(-14.20, 59.78)	0.20	26.59	(-12.29, 65.47)	0.16	-22.43	(-97.15, 52.30)	0.52	4.12	(-62.10, 70.33)	0.89
CO_2	min	0.99	(-2.43, 4.42)	0.54	0.54	(-4.54, 5.61)	0.82	-1.01	(-3.97, 1.95)	0.47	-0.18	(-3.43, 3.06)	0.90	-2.37	(-7.94, 3.20)	0.37	-3.23	(-7.79, 1.32)	0.15
	mean	0.23	(-0.81, 1.27)	0.63	-0.26	(-1.79, 1.26)	0.71	-0.31	(-1.20, 0.58)	0.46	-0.06	(-1.04, 0.92)	0.89	-0.99	(-2.61, 0.62)	0.20	-0.76	(-2.19, 0.67)	0.27
	max	0.09	(-0.41, 0.58)	0.71	-0.07	(-0.80, 0.65)	0.82	-0.21	(-0.62, 0.20)	0.29	-0.11	(-0.57, 0.34)	0.59	-0.23	(-1.04, 0.57)	0.54	-0.28	(-0.98, 0.41)	0.38
	gm	0.24	(-0.91, 1.38)	0.66	-0.33	(-2.00, 1.35)	0.68	-0.34	(-1.32, 0.64)	0.46	-0.07	(-1.15, 1.00)	0.88	-1.19	(-2.94, 0.56)	0.16	-0.89	(-2.45, 0.67)	0.24

Supplementary Table S4. Association between indoor air pollutants and brain-related indicators after correction for cerebral volume (Model 2)

Variable		Cerebral surface area						Cerebral cortex thickness								
		Left hemisphere			Right hemisphere			Left hemisphere average			Right hemisphere average			Overall average		
		estimate	95%CI	p-value	estimate	95%CI	p-value	estimate	95%CI	p-value	estimate	95%CI	p-value	estimate	95%CI	p-value
$PM_{2.5}$	min	-162.54	(-1563.94, 1238.85)	0.80	-235.32	(-1705.95, 1235.30)	0.73	0.00	(-0.04, 0.05)	0.81	0.00	(-0.04, 0.05)	0.83	0.00	(-0.04, 0.05)	0.82
	mean	-33.68	(-200.07, 132.70)	0.66	-21.36	(-197.61, 154.90)	0.79	0.00	(-0.00, 0.01)	0.13	0.00	(-0.00, 0.01)	0.07	0.00	(-0.00, 0.01)	0.08
	max	8.97	(-53.55, 71.49)	0.76	20.44	(-44.12, 85.00)	0.50	0.00	(-0.00, 0.00)	0.44	0.00	(-0.00, 0.00)	0.37	0.00	(-0.00, 0.00)	0.39
	gm	-80.98	(-343.48, 181.51)	0.51	-60.67	(-340.16, 218.82)	0.64	0.01	(-0.00, 0.01)	0.17	0.01	(-0.00, 0.01)	0.05	0.01	(-0.00, 0.01)	0.09
PM_{10}	min	-340.30	(-1373.59, 692.99)	0.48	-369.51	(-1455.20, 716.19)	0.47	0.00	(-0.03, 0.03)	0.94	0.00	(-0.03, 0.03)	0.95	0.00	(-0.03, 0.03)	0.94
	mean	-45.98	(-195.80, 103.85)	0.51	-29.02	(-189.06, 131.02)	0.69	0.00	(-0.00, 0.01)	0.14	0.00	(-0.00, 0.01)	0.08	0.00	(-0.00, 0.01)	0.10
	max	0.40	(-23.06, 23.86)	0.97	2.17	(-22.48, 26.81)	0.85	0.00	(-0.00, 0.00)	0.19	0.00	(-0.00, 0.00)	0.08	0.00	(-0.00, 0.00)	0.12
	gm	-113.38	(-349.60, 122.83)	0.31	-81.28	(-337.41, 174.86)	0.50	0.00	(-0.00, 0.01)	0.19	0.01	(-0.00, 0.01)	0.08	0.01	(-0.00, 0.01)	0.12
CO_2	min	-4.05	(-22.92, 14.81)	0.64	-5.37	(-25.09, 14.35)	0.56	0.00	(-0.00, 0.00)	0.44	0.00	(-0.00, 0.00)	0.20	0.00	(-0.00, 0.00)	0.29
	mean	-1.99	(-7.50, 3.52)	0.44	-2.35	(-8.11, 3.40)	0.38	0.00	(-0.00, 0.00)	0.57	0.00	(-0.00, 0.00)	0.14	0.00	(-0.00, 0.00)	0.30
	max	0.04	(-2.66, 2.73)	0.98	-0.19	(-3.02, 2.64)	0.89	0.00	(-0.00, 0.00)	0.48	0.00	(-0.00, 0.00)	0.12	0.00	(-0.00, 0.00)	0.25
	gm	-2.35	(-8.40, 3.70)	0.41	-2.76	(-9.07, 3.54)	0.35	0.00	(-0.00, 0.00)	0.61	0.00	(-0.00, 0.00)	0.15	0.00	(-0.00, 0.00)	0.32

Supplementary Table S4. Association between indoor air pollutants and brain-related indicators after correction for cerebral volume (Model 2)(Continued)

Variable		Cerebral cortex thickness														
		Left hemisphere frontal lobe			Right hemisphere frontal lobe			Left hemisphere parietal lobe			Right hemisphere parietal lobe			Left hemisphere temporal lobe		
		estimate	95%CI	p-value	estimate	95%CI	p-value	estimate	95%CI	p-value	estimate	95%CI	p-value	estimate	95%CI	p-value
$PM_{2.5}$	min	0.00	(-0.05, 0.05)	0.95	-0.01	(-0.06, 0.04)	0.63	0.00	(-0.05, 0.05)	0.95	0.01	(-0.03, 0.05)	0.56	0.03	(-0.02, 0.08)	0.17
	mean	0.01	(0.00, 0.01)	0.03	0.01	(0.00, 0.01)	0.04	0.00	(-0.00, 0.01)	0.53	0.00	(-0.00, 0.01)	0.20	0.00	(-0.00, 0.01)	0.08
	max	0.00	(-0.00, 0.00)	0.16	0.00	(-0.00, 0.00)	0.18	0.00	(-0.00, 0.00)	0.68	0.00	(-0.00, 0.00)	0.43	0.00	(-0.00, 0.00)	0.41
	gm	0.01	(0.00, 0.02)	0.04	0.01	(0.00, 0.02)	0.03	0.00	(-0.01, 0.01)	0.63	0.00	(-0.00, 0.01)	0.15	0.01	(-0.00, 0.02)	0.06
PM_{10}	min	-0.01	(-0.04, 0.03)	0.72	-0.01	(-0.05, 0.03)	0.47	-0.01	(-0.05, 0.03)	0.65	0.00	(-0.03, 0.03)	0.80	0.02	(-0.02, 0.06)	0.24
	mean	0.00	(0.00, 0.01)	0.02	0.00	(0.00, 0.01)	0.04	0.00	(-0.00, 0.01)	0.63	0.00	(-0.00, 0.01)	0.26	0.00	(-0.00, 0.01)	0.07
	max	0.00	(-0.00, 0.00)	0.06	0.00	(-0.00, 0.00)	0.06	0.00	(-0.00, 0.00)	0.50	0.00	(-0.00, 0.00)	0.11	0.00	(-0.00, 0.00)	0.09
	gm	0.01	(0.00, 0.01)	0.05	0.01	(-0.00, 0.02)	0.05	0.00	(-0.01, 0.01)	0.78	0.00	(-0.00, 0.01)	0.24	0.01	(-0.00, 0.02)	0.05
CO_2	min	0.00	(-0.00, 0.00)	0.70	0.00	(-0.00, 0.00)	0.20	0.00	(-0.00, 0.00)	0.29	0.00	(-0.00, 0.00)	0.26	0.00	(-0.00, 0.00)	0.71
	mean	0.00	(-0.00, 0.00)	0.57	0.00	(-0.00, 0.00)	0.06	0.00	(-0.00, 0.00)	0.59	0.00	(-0.00, 0.00)	0.25	0.00	(-0.00, 0.00)	0.62
	max	0.00	(-0.00, 0.00)	0.45	0.00	(0.00, 0.00)	0.05	0.00	(-0.00, 0.00)	0.35	0.00	(-0.00, 0.00)	0.17	0.00	(-0.00, 0.00)	0.67
	gm	0.00	(-0.00, 0.00)	0.64	0.00	(-0.00, 0.00)	0.08	0.00	(-0.00, 0.00)	0.61	0.00	(-0.00, 0.00)	0.25	0.00	(-0.00, 0.00)	0.63

Supplementary Table S4. Association between indoor air pollutants and brain-related indicators after correction for cerebral volume (Model 2)(Continued)

Variable		Cerebral cortex thickness														
		Right hemisphere temporal lobe			Left hemisphere occipital lobe			Right hemisphere occipital lobe			Left hemisphere cingulate gyrus			Right hemisphere cingulate gyrus		
		estimate	95%CI	p-value	estimate	95%CI	p-value	estimate	95%CI	p-value	estimate	95%CI	p-value	estimate	95%CI	p-value
$PM_{2.5}$	min	0.02	(-0.03, 0.08)	0.38	0.01	(-0.03, 0.05)	0.63	-0.01	(-0.04, 0.03)	0.70	-0.03	(-0.13, 0.06)	0.45	0.05	(-0.02, 0.12)	0.17
	mean	0.00	(-0.00, 0.01)	0.12	0.00	(-0.01, 0.00)	0.64	0.00	(-0.01, 0.00)	0.32	0.00	(-0.01, 0.01)	0.74	0.01	(-0.00, 0.01)	0.23
	max	0.00	(-0.00, 0.00)	0.59	0.00	(-0.00, 0.00)	0.46	0.00	(-0.00, 0.00)	0.11	0.00	(-0.01, 0.00)	0.22	0.00	(-0.00, 0.00)	0.82
	gm	0.01	(-0.00, 0.02)	0.10	0.00	(-0.01, 0.01)	0.67	0.00	(-0.01, 0.00)	0.30	0.00	(-0.02, 0.02)	0.80	0.01	(-0.01, 0.02)	0.22
PM_{10}	min	0.01	(-0.03, 0.05)	0.50	0.01	(-0.02, 0.03)	0.60	-0.01	(-0.03, 0.02)	0.67	-0.02	(-0.09, 0.05)	0.47	0.04	(-0.02, 0.09)	0.16
	mean	0.00	(-0.00, 0.01)	0.12	0.00	(-0.00, 0.00)	0.68	0.00	(-0.01, 0.00)	0.29	0.00	(-0.01, 0.01)	0.64	0.00	(-0.00, 0.01)	0.21
	max	0.00	(-0.00, 0.00)	0.16	0.00	(-0.00, 0.00)	0.38	0.00	(-0.00, 0.00)	0.27	0.00	(-0.00, 0.00)	0.84	0.00	(-0.00, 0.00)	0.37
	gm	0.01	(-0.00, 0.02)	0.11	0.00	(-0.01, 0.01)	0.75	0.00	(-0.01, 0.00)	0.28	0.00	(-0.01, 0.02)	0.68	0.01	(-0.00, 0.02)	0.18
CO_2	min	0.00	(-0.00, 0.00)	0.64	0.00	(-0.00, 0.00)	0.97	0.00	(-0.00, 0.00)	0.33	0.00	(-0.00, 0.00)	0.23	0.00	(-0.00, 0.00)	0.62
	mean	0.00	(-0.00, 0.00)	0.38	0.00	(-0.00, 0.00)	0.87	0.00	(-0.00, 0.00)	0.64	0.00	(-0.00, 0.00)	0.41	0.00	(-0.00, 0.00)	0.71
	max	0.00	(-0.00, 0.00)	0.39	0.00	(-0.00, 0.00)	0.60	0.00	(-0.00, 0.00)	0.69	0.00	(-0.00, 0.00)	0.54	0.00	(-0.00, 0.00)	0.39
	gm	0.00	(-0.00, 0.00)	0.39	0.00	(-0.00, 0.00)	0.94	0.00	(-0.00, 0.00)	0.60	0.00	(-0.00, 0.00)	0.43	0.00	(-0.00, 0.00)	0.74

Supplementary Table S4. Association between indoor air pollutants and brain-related indicators after correction for cerebral volume (Model 2)(Continued)

Variable		Cerebral cortex thickness						Subcortical volume								
		Left hemisphere insular lobe			Right hemisphere insular lobe			Left hemisphere nucleus accumbens			Right hemisphere nucleus accumbens			Left hemisphere amygdaloid body		
		estimate	95%CI	p-value	estimate	95%CI	p-value	estimate	95%CI	p-value	estimate	95%CI	p-value	estimate	95%CI	p-value
$PM_{2.5}$	min	-0.03	(-0.13, 0.07)	0.50	-0.02	(-0.18, 0.14)	0.80	31.06	(2.64, 59.47)	0.04	7.96	(-38.22, 54.14)	0.71	-51.69	(-138.99, 35.61)	0.22
	mean	0.01	(-0.00, 0.02)	0.11	0.01	(-0.01, 0.03)	0.27	0.92	(-3.32, 5.16)	0.64	2.11	(-3.25, 7.47)	0.40	1.89	(-9.34, 13.12)	0.72
	max	0.00	(-0.00, 0.00)	0.97	0.00	(-0.01, 0.01)	0.78	0.54	(-1.02, 2.10)	0.46	0.57	(-1.46, 2.61)	0.54	1.13	(-3.03, 5.28)	0.56
	gm	0.01	(-0.01, 0.03)	0.24	0.02	(-0.01, 0.05)	0.16	1.47	(-5.30, 8.25)	0.64	3.23	(-5.36, 11.82)	0.42	-0.06	(-18.13, 18.01)	0.99
PM_{10}	min	-0.03	(-0.11, 0.04)	0.30	-0.02	(-0.14, 0.10)	0.72	18.76	(-4.85, 42.37)	0.11	0.47	(-34.62, 35.57)	0.98	-52.33	(-113.42, 8.77)	0.09
	mean	0.01	(-0.00, 0.02)	0.13	0.01	(-0.01, 0.02)	0.29	0.57	(-3.32, 4.46)	0.75	1.80	(-3.10, 6.71)	0.43	1.31	(-8.96, 11.58)	0.78
	max	0.00	(-0.00, 0.00)	0.33	0.00	(-0.00, 0.00)	0.22	0.32	(-0.23, 0.87)	0.23	0.35	(-0.38, 1.09)	0.31	-0.06	(-1.64, 1.52)	0.94
	gm	0.01	(-0.01, 0.02)	0.33	0.02	(-0.01, 0.04)	0.22	0.87	(-5.46, 7.20)	0.77	2.56	(-5.49, 10.61)	0.49	-1.57	(-18.31, 15.17)	0.84
CO_2	min	0.00	(-0.00, 0.00)	0.89	0.00	(-0.00, 0.00)	0.35	0.13	(-0.35, 0.61)	0.56	0.11	(-0.52, 0.74)	0.71	-0.66	(-1.86, 0.54)	0.25
	mean	0.00	(-0.00, 0.00)	0.84	0.00	(-0.00, 0.00)	0.22	-0.02	(-0.16, 0.12)	0.76	-0.02	(-0.21, 0.16)	0.78	-0.15	(-0.52, 0.21)	0.38
	max	0.00	(-0.00, 0.00)	0.66	0.00	(-0.00, 0.00)	0.25	0.01	(-0.06, 0.07)	0.86	0.00	(-0.09, 0.09)	0.98	-0.03	(-0.21, 0.15)	0.73
	gm	0.00	(-0.00, 0.00)	0.93	0.00	(-0.00, 0.00)	0.24	-0.02	(-0.18, 0.14)	0.74	-0.03	(-0.24, 0.18)	0.76	-0.18	(-0.59, 0.22)	0.33

Supplementary Table S4. Association between indoor air pollutants and brain-related indicators after correction for cerebral volume (Model 2)(Continued)

Variable		Subcortical volume														
		Right hemisphere amygdaloid body			Left hemisphere hippocampus			Right hemisphere hippocampus			Left hemisphere globus pallidus			Right hemisphere globus pallidus		
		estimate	95%CI	p-value	estimate	95%CI	p-value	estimate	95%CI	p-value	estimate	95%CI	p-value	estimate	95%CI	p-value
$PM_{2.5}$	min	-28.22	(-124.00, 67.56)	0.53	-35.89	(-257.32,185.55)	0.73	-34.69	(-244.34, 174.96)	0.72	33.57	(-77.21, 144.35)	0.51	2.59	(-112.33, 117.50)	0.96
	mean	0.44	(-11.25, 12.13)	0.93	-3.29	(-29.83, 23.25)	0.79	-0.20	(-25.43, 25.02)	0.99	5.42	(-7.57, 18.41)	0.37	8.38	(-4.02, 20.79)	0.16
	max	0.13	(-4.24, 4.50)	0.95	0.77	(-9.18, 10.72)	0.87	1.38	(-8.01, 10.76)	0.75	2.13	(-2.70, 6.97)	0.35	3.83	(-0.53, 8.20)	0.08
	gm	-1.55	(-20.21, 17.11)	0.86	-9.59	(-51.63, 32.45)	0.62	-5.25	(-45.40, 34.91)	0.78	6.56	(-14.58, 27.71)	0.50	10.83	(-9.75, 31.42)	0.27
PM_{10}	min	-34.73	(-104.35, 34.89)	0.29	-53.05	(-216.98, 110.88)	0.49	-46.43	(-202.27, 109.40)	0.52	17.63	(-66.92, 102.17)	0.65	-11.15	(-97.50, 75.20)	0.78
	mean	-0.20	(-10.86, 10.47)	0.97	-3.33	(-27.52, 20.85)	0.76	0.21	(-22.80, 23.22)	0.98	5.46	(-6.27, 17.19)	0.32	7.70	(-3.60, 18.99)	0.16
	max	-0.26	(-1.88, 1.36)	0.73	-0.31	(-4.02, 3.40)	0.85	-0.44	(-3.95, 3.07)	0.79	0.85	(-0.94, 2.64)	0.31	1.50	(-0.11, 3.10)	0.06
	gm	-3.13	(-20.34, 14.08)	0.69	-10.43	(-49.27, 28.40)	0.56	-4.67	(-41.96, 32.62)	0.79	7.41	(-11.99, 26.81)	0.41	9.88	(-9.28, 29.04)	0.28
CO_2	min	-0.76	(-1.97, 0.46)	0.20	-2.29	(-4.85, 0.26)	0.07	-2.88	(-4.90, -0.86)	**0.01**	0.27	(-1.26, 1.79)	0.70	0.53	(-0.99, 2.04)	0.46
	mean	-0.18	(-0.56, 0.19)	0.30	-0.77	(-1.49, -0.04)	**0.04**	-0.77	(-1.43, -0.10)	**0.03**	-0.06	(-0.52, 0.39)	0.76	0.03	(-0.44, 0.49)	0.90
	max	-0.05	(-0.24, 0.13)	0.55	-0.25	(-0.64, 0.14)	0.19	-0.28	(-0.63, 0.07)	0.11	-0.01	(-0.22, 0.21)	0.94	0.07	(-0.15, 0.28)	0.51
	gm	-0.21	(-0.62, 0.20)	0.27	-0.88	(-1.66, -0.11)	**0.03**	-0.88	(-1.59, -0.17)	**0.02**	-0.08	(-0.58, 0.42)	0.73	0.01	(-0.51, 0.52)	0.98

Supplementary Table S4. Association between indoor air pollutants and brain-related indicators after correction for cerebral volume (Model 2)(Continued)

Variable		Subcortical volume																	
		Left hemisphere putamen			Right hemisphere putamen			Left hemisphere caudate nucleus			Right hemisphere caudate nucleus			Left hemisphere thalamus			Right hemisphere thalamus		
		estimate	95%CI	p-value	estimate	95%CI	p-value	estimate	95%CI	p-value	estimate	95%CI	p-value	estimate	95%CI	p-value	estimate	95%CI	p-value
$PM_{2.5}$	min	-33.74	(-256.84, 189.37)	0.74	46.64	(-352.39, 445.68)	0.80	53.04	(-139.03, 245.12)	0.55	-48.34	(-223.05, 126.37)	0.55	18.41	(-339.20, 376.02)	0.91	31.10	(-193.24, 255.44)	0.76
	mean	5.91	(-20.59, 32.40)	0.63	17.40	(-28.87, 63.66)	0.42	12.64	(-8.98, 34.27)	0.22	14.40	(-4.30, 33.10)	0.12	-17.26	(-58.27, 23.75)	0.37	-6.99	(-33.48, 19.50)	0.57
	max	0.62	(-9.40, 10.64)	0.89	4.54	(-13.07, 22.15)	0.58	2.30	(-6.30, 10.89)	0.56	2.14	(-5.67, 9.95)	0.55	-2.20	(-18.12, 13.71)	0.76	1.79	(-8.21, 11.78)	0.70
	gm	9.94	(-32.35, 52.23)	0.61	16.47	(-59.14, 92.08)	0.64	15.24	(-20.57, 51.04)	0.37	17.68	(-13.96, 49.32)	0.24	-34.90	(-98.69, 28.89)	0.25	-13.35	(-55.37, 28.68)	0.50
PM_{10}	min	-27.99	(-196.11, 140.12)	0.72	7.89	(-294.12, 309.89)	0.95	32.28	(-113.57, 178.14)	0.63	-38.10	(-169.67, 93.47)	0.53	-30.06	(-299.19, 239.07)	0.81	7.05	(-162.93, 177.02)	0.93
	mean	7.51	(-16.37, 31.39)	0.50	17.82	(-23.98, 59.62)	0.36	12.70	(-6.67, 32.06)	0.17	14.39	(-2.16, 30.93)	0.08	-17.62	(-54.60, 19.36)	0.31	-5.83	(-30.06, 18.40)	0.60
	max	1.13	(-2.53, 4.79)	0.51	2.00	(-4.53, 8.53)	0.51	0.62	(-2.62, 3.85)	0.68	0.67	(-2.26, 3.61)	0.62	-0.87	(-6.81, 5.07)	0.75	-0.62	(-4.35, 3.12)	0.72
	gm	13.71	(-24.89, 52.31)	0.45	20.31	(-49.24, 89.86)	0.53	17.15	(-15.37, 49.68)	0.27	19.10	(-9.46, 47.65)	0.17	-36.04	(-94.20, 22.12)	0.20	-10.44	(-49.73, 28.85)	0.57
CO_2	min	1.72	(-1.08, 4.51)	0.20	0.86	(-4.53, 6.26)	0.73	-0.47	(-3.10, 2.17)	0.70	0.58	(-1.80, 2.96)	0.60	-1.28	(-6.05, 3.49)	0.56	-2.01	(-4.72, 0.70)	0.13
	mean	0.37	(-0.49, 1.24)	0.36	-0.21	(-1.82, 1.41)	0.78	-0.20	(-0.98, 0.58)	0.59	0.09	(-0.62, 0.81)	0.78	-0.77	(-2.11, 0.57)	0.23	-0.50	(-1.34, 0.34)	0.21
	max	0.17	(-0.25, 0.58)	0.39	-0.04	(-0.81, 0.72)	0.91	-0.15	(-0.51, 0.22)	0.39	-0.03	(-0.37, 0.31)	0.86	-0.10	(-0.78, 0.58)	0.75	-0.13	(-0.56, 0.29)	0.49
	gm	0.41	(-0.55, 1.37)	0.36	-0.26	(-2.03, 1.52)	0.75	-0.20	(-1.06, 0.66)	0.61	0.12	(-0.67, 0.91)	0.75	-0.92	(-2.38, 0.54)	0.19	-0.57	(-1.49, 0.35)	0.19

Supplementary Table S5. Association between indoor air pollutants and brain-related indicators without adjustment for cerebral volume (Model 3)

Variable		Cerebral surface area						Cerebral cortex thickness								
		Left hemisphere			Right hemisphere			Left hemisphere average			Right hemisphere average			Overall average		
		estimate	95%CI	p-value	estimate	95%CI	p-value	estimate	95%CI	p-value	estimate	95%CI	p-value	estimate	95%CI	p-value
$PM_{2.5}$	mean	-2211.81	(-14321.03, 9897.40)	0.70	-1839.53	(-13897.75, 10218.69)	0.74	-0.01	(-0.13, 0.11)	0.83	-0.01	(-0.13, 0.10)	0.79	-0.01	(-0.13, 0.10)	0.80
	gm	-2004.41	(-13543.74, 9534.92)	0.71	-2156.47	(-13608.87, 9295.93)	0.69	0.08	(-0.02, 0.18)	0.12	0.06	(-0.04, 0.16)	0.18	0.07	(-0.03, 0.17)	0.13
PM_{10}	mean	-4023.33	(-17351.94, 9305.28)	0.52	-3512.66	(-16816.47, 9791.14)	0.57	0.08	(-0.04, 0.20)	0.17	0.06	(-0.06, 0.18)	0.31	0.07	(-0.05, 0.19)	0.22
	gm	-750.14	(-11809.59, 10309.31)	0.88	-362.39	(-11359.62, 10634.84)	0.94	-0.03	(-0.14, 0.07)	0.49	-0.04	(-0.14, 0.06)	0.38	-0.04	(-0.14, 0.06)	0.42
CO_2	mean	-688.24	(-9432.01, 8055.54)	0.87	-1222.95	(-9884.72, 7438.83)	0.76	0.01	(-0.08, 0.09)	0.83	0.05	(-0.02, 0.12)	0.15	0.03	(-0.05, 0.11)	0.42
	max	-7534.60	(-23899.46, 8830.26)	0.33	-7139.80	(-23468.53, 9188.93)	0.36	0.08	(-0.08, 0.24)	0.28	0.09	(-0.06, 0.24)	0.20	0.09	(-0.06, 0.23)	0.22
	gm	-1588.25	(-9908.90, 6732.39)	0.68	-2050.39	(-10272.06, 6171.29)	0.59	0.00	(-0.09, 0.08)	0.94	0.03	(-0.04, 0.11)	0.34	0.02	(-0.06, 0.09)	0.67

Supplementary Table S5. Association between indoor air pollutants and brain-related indicators without adjustment for cerebral volume (Model 3)(Continued)

Variable		Cerebral cortex thickness														
		Left hemisphere frontal lobe			Right hemisphere frontal lobe			Left hemisphere parietal lobe			Right hemisphere parietal lobe			Left hemisphere temporal lobe		
		estimate	95%CI	p-value	estimate	95%CI	p-value	estimate	95%CI	p-value	estimate	95%CI	p-value	estimate	95%CI	p-value
$PM_{2.5}$	mean	0.01	(-0.13, 0.15)	0.93	-0.02	(-0.17, 0.12)	0.72	-0.03	(-0.17, 0.10)	0.62	-0.01	(-0.11, 0.10)	0.91	0.04	(-0.11, 0.19)	0.56
	gm	0.08	(-0.04, 0.21)	0.16	0.04	(-0.10, 0.17)	0.55	0.04	(-0.08, 0.17)	0.46	0.06	(-0.04, 0.15)	0.21	0.15	(0.05, 0.26)	0.01
PM_{10}	mean	0.10	(-0.04, 0.24)	0.15	0.06	(-0.09, 0.21)	0.40	0.03	(-0.12, 0.18)	0.72	0.03	(-0.09, 0.15)	0.57	0.12	(-0.03, 0.27)	0.11
	gm	-0.01	(-0.13, 0.12)	0.91	-0.06	(-0.18, 0.07)	0.33	-0.08	(-0.19, 0.04)	0.16	-0.03	(-0.13, 0.06)	0.46	0.03	(-0.11, 0.17)	0.64
CO_2	mean	0.00	(-0.10, 0.10)	0.97	0.07	(-0.02, 0.16)	0.13	0.01	(-0.09, 0.10)	0.90	0.04	(-0.03, 0.12)	0.19	0.02	(-0.09, 0.13)	0.66
	max	0.11	(-0.07, 0.30)	0.19	0.12	(-0.07, 0.30)	0.19	0.11	(-0.06, 0.29)	0.19	0.12	(-0.01, 0.25)	0.07	0.07	(-0.13, 0.28)	0.45
	gm	-0.01	(-0.11, 0.08)	0.79	0.05	(-0.05, 0.14)	0.30	0.00	(-0.09, 0.09)	0.99	0.03	(-0.04, 0.10)	0.32	0.01	(-0.10, 0.11)	0.89

Supplementary Table S5. Association between indoor air pollutants and brain-related indicators without adjustment for cerebral volume (Model 3)(Continued)

Variable		Cerebral cortex thickness														
		Right hemisphere temporal lobe			Left hemisphere occipital lobe			Right hemisphere occipital lobe			Left hemisphere cingulate gyrus			Right hemisphere cingulate gyrus		
		estimate	95%CI	p-value	estimate	95%CI	p-value	estimate	95%CI	p-value	estimate	95%CI	p-value	estimate	95%CI	p-value
$PM_{2.5}$	mean	-0.01	(-0.15, 0.14)	0.90	-0.04	(-0.13, 0.05)	0.37	-0.04	(-0.13, 0.05)	0.36	-0.02	(-0.29, 0.26)	0.89	0.16	(-0.03, 0.35)	0.09
	gm	0.12	(0.00, 0.23)	0.04	0.01	(-0.08, 0.10)	0.81	-0.02	(-0.11, 0.07)	0.69	0.11	(-0.14, 0.36)	0.35	0.19	(0.03, 0.36)	0.02
PM_{10}	mean	0.09	(-0.06, 0.24)	0.22	0.00	(-0.11, 0.11)	0.97	-0.03	(-0.14, 0.07)	0.53	0.25	(-0.01, 0.51)	0.06	0.15	(-0.07, 0.37)	0.17
	gm	-0.01	(-0.14, 0.13)	0.92	-0.04	(-0.13, 0.04)	0.30	-0.07	(-0.15, 0.00)	0.05	-0.05	(-0.30, 0.19)	0.64	0.10	(-0.08, 0.29)	0.24
CO_2	mean	0.06	(-0.04, 0.16)	0.21	0.00	(-0.07, 0.07)	0.99	0.01	(-0.06, 0.08)	0.85	0.04	(-0.15, 0.24)	0.62	-0.06	(-0.21, 0.09)	0.38
	max	0.02	(-0.19, 0.22)	0.86	-0.05	(-0.19, 0.08)	0.41	0.04	(-0.10, 0.17)	0.55	0.26	(-0.09, 0.60)	0.13	0.02	(-0.29, 0.32)	0.91
	gm	0.03	(-0.07, 0.13)	0.48	0.00	(-0.07, 0.06)	0.93	0.00	(-0.06, 0.07)	0.90	0.02	(-0.17, 0.21)	0.81	-0.05	(-0.20, 0.09)	0.43

Supplementary Table S5**.** Association between indoor air pollutants and brain-related indicators without adjustment for cerebral volume (Model 3)(Continued)

Variable		Cerebral cortex thickness						Subcortical volume								
		Left hemisphere insular lobe			Right hemisphere insular lobe			Left hemisphere nucleus accumbens			Right hemisphere nucleus accumbens			Left hemisphere amygdaloid body		
		estimate	95%CI	p-value	estimate	95%CI	p-value	estimate	95%CI	p-value	estimate	95%CI	p-value	estimate	95%CI	p-value
$PM_{2.5}$	mean	-0.07	(-0.34, 0.20)	0.59	-0.14	(-0.54, 0.27)	0.48	53.71	(-32.29, 139.72)	0.20	-37.10	(-165.96, 91.75)	0.54	-283.24	(-496.78, -69.71)	0.01
	gm	0.16	(-0.08, 0.40)	0.18	0.09	(-0.30, 0.48)	0.62	98.18	(38.04, 158.31)	0.00	69.61	(-46.46, 185.69)	0.21	-79.35	(-345.08, 186.39)	0.52
PM_{10}	mean	0.18	(-0.10, 0.46)	0.19	0.18	(-0.27, 0.63)	0.40	46.55	(-52.50, 145.60)	0.32	91.87	(-41.01, 224.75)	0.16	5.54	(-311.46, 322.55)	0.97
	gm	-0.05	(-0.30, 0.20)	0.66	-0.19	(-0.55, 0.16)	0.26	40.06	(-40.14, 120.27)	0.30	-26.02	(-143.83, 91.78)	0.64	-157.74	(-393.76, 78.28)	0.17
CO_2	mean	0.00	(-0.19, 0.20)	0.97	0.20	(-0.07, 0.47)	0.13	-1.64	(-68.47, 65.18)	0.96	-2.96	(-97.10, 91.19)	0.95	-46.97	(-248.78, 154.84)	0.62
	max	0.23	(-0.13, 0.58)	0.19	0.45	(-0.06, 0.95)	0.08	-6.26	(-136.81, 124.28)	0.92	-3.76	(-187.78, 180.26)	0.96	-122.21	(-512.96, 268.55)	0.51
	gm	-0.03	(-0.22, 0.16)	0.72	0.12	(-0.16, 0.39)	0.37	2.85	(-61.14, 66.84)	0.92	-18.88	(-108.21, 70.45)	0.65	-85.54	(-272.73, 101.65)	0.34

Supplementary Table S5**.** Association between indoor air pollutants and brain-related indicators without adjustment for cerebral volume (Model 3)(Continued)

Variable		Subcortical volume														
		Right hemisphere amygdaloid body			Left hemisphere hippocampus			Right hemisphere hippocampus			Left hemisphere globus pallidus			Right hemisphere globus pallidus		
		estimate	95%CI	p-value	estimate	95%CI	p-value	estimate	95%CI	p-value	estimate	95%CI	p-value	estimate	95%CI	p-value
$PM_{2.5}$	mean	-292.37	(-549.47, -35.27)	**0.03**	-506.97	(-1139.92, 125.98)	0.11	-544.55	(-1088.22, -0.88)	**0.05**	87.05	(-371.89, 545.98)	0.68	136.39	(-253.95, 526.72)	0.46
	gm	-67.80	(-371.21, 235.60)	0.63	-13.71	(-696.25, 668.83)	0.97	-21.78	(-643.27, 599.71)	0.94	177.55	(-246.87, 601.98)	0.38	149.45	(-219.00, 517.91)	0.39
PM_{10}	mean	-88.55	(-442.65, 265.55)	0.59	167.43	(-623.62, 958.48)	0.65	161.65	(-557.90, 881.20)	0.63	175.31	(-326.87, 677.48)	0.46	194.91	(-232.48, 622.30)	0.34
	gm	-194.88	(-457.05, 67.28)	0.13	-137.03	(-781.21, 507.16)	0.65	-102.27	(-690.86, 486.32)	0.71	125.14	(-286.38, 536.65)	0.52	137.43	(-214.61, 489.47)	0.41
CO_2	mean	-69.34	(-295.92, 157.23)	0.51	-256.89	(-742.36, 228.58)	0.27	-247.63	(-686.46, 191.20)	0.24	-62.14	(-391.60, 267.31)	0.69	-44.25	(-330.36, 241.87)	0.74
	max	-86.91	(-535.07, 361.24)	0.68	-340.08	(-1320.00, 639.84)	0.46	-546.80	(-1387.64, 294.04)	0.18	-499.96	(-1057.61, 57.70)	0.07	-144.21	(-698.11, 409.70)	0.58
	gm	-120.05	(-326.69, 86.59)	0.23	-345.74	(-781.95, 90.47)	0.11	-350.17	(-734.20, 33.87)	0.07	-96.89	(-408.37, 214.59)	0.51	-54.67	(-327.78, 218.45)	0.67

Supplementary Table S5**.** Association between indoor air pollutants and brain-related indicators without adjustment for cerebral volume (Model 3)(Continued)

Variable		Subcortical volume																	
		Left hemisphere putamen			Right hemisphere putamen			Left hemisphere caudate nucleus			Right hemisphere caudate nucleus			Left hemisphere thalamus			Right hemisphere thalamus		
		estimate	95%CI	p-value	estimate	95%CI	p-value	estimate	95%CI	p-value	estimate	95%CI	p-value	estimate	95%CI	p-value	estimate	95%CI	p-value
$PM_{2.5}$	mean	-100.93	(-822.12, 620.27)	0.76	296.76	(-741.84, 1335.36)	0.54	61.22	(-567.92, 690.36)	0.83	50.14	(-622.61, 722.89)	0.87	-359.05	(-1537.26, 819.16)	0.52	-368.90	(-1385.36, 647.56)	0.44
	gm	143.18	(-539.97, 826.34)	0.65	654.27	(-253.94, 1562.48)	0.14	244.52	(-333.50, 822.54)	0.37	24.01	(-617.22, 665.24)	0.94	96.85	(-1046.04, 1239.74)	0.86	-134.46	(-1126.09, 857.16)	0.77
PM_{10}	mean	447.73	(-302.76, 1198.21)	0.22	778.87	(-279.73, 1837.48)	0.13	126.23	(-571.34, 823.80)	0.70	54.77	(-694.98, 804.53)	0.88	-48.91	(-1388.10, 1290.29)	0.94	-283.74	(-1433.57, 866.10)	0.60
	gm	-123.05	(-775.33, 529.22)	0.69	300.94	(-637.46, 1239.34)	0.49	126.47	(-439.53, 692.48)	0.63	-2.24	(-613.57, 609.09)	0.99	-122.10	(-1210.07, 965.86)	0.81	-106.39	(-1052.68, 839.89)	0.81
CO_2	mean	119.02	(-394.85, 632.89)	0.62	-303.73	(-1035.27, 427.81)	0.38	-203.27	(-635.24, 228.69)	0.32	-158.02	(-630.01, 313.96)	0.48	-278.56	(-1121.37, 564.25)	0.48	-214.73	(-951.57, 522.10)	0.53
	max	-202.40	(-1209.56, 804.76)	0.67	-492.55	(-1939.03, 953.94)	0.47	-509.92	(-1327.05, 307.20)	0.20	-350.25	(-1266.13, 565.64)	0.42	-774.52	(-2380.59, 831.54)	0.31	-943.12	(-2269.60, 383.35)	0.15
	gm	20.78	(-477.05, 518.61)	0.93	-296.87	(-996.52, 402.78)	0.37	-230.41	(-636.04, 175.22)	0.24	-191.25	(-636.66, 254.17)	0.36	-371.11	(-1160.09, 417.87)	0.32	-335.54	(-1019.08, 348.01)	0.30

Supplementary Table S6**.** Association between indoor air pollutants and brain-related indicators after correction for cerebral volume (Model 4)

Variable		Cerebral surface area						Cerebral cortex thickness								
		Left hemisphere			Right hemisphere			Left hemisphere average			Right hemisphere average			Overall average		
		estimate	95%CI	p-value	estimate	95%CI	p-value	estimate	95%CI	p-value	estimate	95%CI	p-value	estimate	95%CI	p-value
$PM_{2.5}$	mean	-1244.94	(-5025.71, 2535.82)	0.48	-883.57	(-4920.96, 3153.81)	0.64	-0.02	(-0.13, 0.10)	0.78	-0.02	(-0.13, 0.10)	0.78	-0.02	(-0.13, 0.10)	0.77
	gm	-211.24	(-3920.07, 3497.59)	0.90	-387.56	(-4284.84, 3509.72)	0.83	0.07	(-0.03, 0.17)	0.15	0.06	(-0.04, 0.17)	0.22	0.07	(-0.03, 0.17)	0.17
PM_{10}	mean	-483.87	(-4862.49, 3894.76)	0.81	-1.25	(-4623.87, 4621.38)	1.00	0.07	(-0.06, 0.20)	0.24	0.05	(-0.07, 0.18)	0.37	0.06	(-0.06, 0.19)	0.29
	gm	-703.31	(-4185.36, 2778.75)	0.66	-316.13	(-4011.36, 3379.09)	0.85	-0.03	(-0.14, 0.07)	0.48	-0.04	(-0.14, 0.06)	0.40	-0.04	(-0.14, 0.06)	0.43
CO_2	mean	-598.40	(-3348.00, 2151.21)	0.64	-1134.25	(-3950.96, 1682.46)	0.39	0.01	(-0.08, 0.09)	0.84	0.05	(-0.03, 0.13)	0.17	0.03	(-0.05, 0.11)	0.43
	max	4208.94	(-1082.65, 9500.54)	0.11	4532.25	(-996.41, 10060.90)	0.10	0.05	(-0.13, 0.24)	0.53	0.09	(-0.08, 0.27)	0.26	0.07	(-0.10, 0.25)	0.37
	gm	-243.73	(-2919.59, 2432.14)	0.84	-728.59	(-3503.76, 2046.57)	0.57	-0.01	(-0.09, 0.07)	0.84	0.03	(-0.05, 0.11)	0.39	0.01	(-0.07, 0.09)	0.74

Supplementary Table S6**.** Association between indoor air pollutants and brain-related indicators after correction for cerebral volume (Model 4)(Continued)

Variable		Cerebral cortex thickness														
		Left hemisphere frontal lobe			Right hemisphere frontal lobe			Left hemisphere parietal lobe			Right hemisphere parietal lobe			Left hemisphere temporal lobe		
		estimate	95%CI	p-value	estimate	95%CI	p-value	estimate	95%CI	p-value	estimate	95%CI	p-value	estimate	95%CI	p-value
$PM_{2.5}$	mean	0.00	(-0.14, 0.14)	0.98	-0.03	(-0.17, 0.12)	0.69	-0.03	(-0.17, 0.11)	0.62	-0.01	(-0.12, 0.11)	0.91	0.04	(-0.11, 0.18)	0.59
	gm	0.08	(-0.04, 0.20)	0.19	0.03	(-0.11, 0.17)	0.61	0.04	(-0.09, 0.18)	0.50	0.06	(-0.04, 0.16)	0.23	0.14	(0.04, 0.24)	0.01
PM_{10}	mean	0.09	(-0.06, 0.23)	0.21	0.05	(-0.11, 0.22)	0.48	0.02	(-0.14, 0.18)	0.77	0.03	(-0.09, 0.16)	0.59	0.10	(-0.05, 0.26)	0.16
	gm	-0.01	(-0.13, 0.12)	0.90	-0.06	(-0.19, 0.07)	0.34	-0.08	(-0.19, 0.04)	0.18	-0.03	(-0.13, 0.07)	0.48	0.03	(-0.10, 0.16)	0.63
CO_2	mean	0.00	(-0.10, 0.10)	0.98	0.07	(-0.03, 0.16)	0.14	0.01	(-0.10, 0.11)	0.91	0.04	(-0.03, 0.12)	0.22	0.02	(-0.08, 0.13)	0.66
	max	0.08	(-0.13, 0.29)	0.42	0.11	(-0.11, 0.33)	0.28	0.12	(-0.09, 0.33)	0.22	0.14	(-0.00, 0.29)	0.05	0.02	(-0.21, 0.26)	0.83
	gm	-0.02	(-0.11, 0.08)	0.68	0.04	(-0.05, 0.14)	0.35	0.00	(-0.10, 0.10)	0.97	0.03	(-0.04, 0.11)	0.34	0.00	(-0.10, 0.10)	1.00

Supplementary Table S6**.** Association between indoor air pollutants and brain-related indicators after correction for cerebral volume (Model 4)(Continued)

Variable		Cerebral cortex thickness														
		Right hemisphere temporal lobe			Left hemisphere occipital lobe			Right hemisphere occipital lobe			Left hemisphere cingulate gyrus			Right hemisphere cingulate gyrus		
		estimate	95%CI	p-value	estimate	95%CI	p-value	estimate	95%CI	p-value	estimate	95%CI	p-value	estimate	95%CI	p-value
$PM_{2.5}$	mean	-0.01	(-0.16, 0.14)	0.88	-0.04	(-0.14, 0.06)	0.38	-0.04	(-0.14, 0.06)	0.39	-0.03	(-0.29, 0.24)	0.83	0.16	(-0.03, 0.34)	0.10
	gm	0.12	(-0.01, 0.24)	0.06	0.01	(-0.09, 0.11)	0.86	-0.02	(-0.11, 0.08)	0.72	0.09	(-0.15, 0.34)	0.41	0.19	(0.02, 0.35)	0.03
PM_{10}	mean	0.08	(-0.08, 0.25)	0.27	0.00	(-0.12, 0.11)	0.96	-0.03	(-0.15, 0.08)	0.56	0.22	(-0.04, 0.48)	0.08	0.13	(-0.10, 0.36)	0.23
	gm	-0.01	(-0.15, 0.13)	0.92	-0.04	(-0.13, 0.05)	0.32	-0.07	(-0.15, 0.00)	0.06	-0.05	(-0.29, 0.18)	0.62	0.10	(-0.08, 0.29)	0.24
CO_2	mean	0.06	(-0.04, 0.16)	0.23	0.00	(-0.07, 0.07)	0.99	0.01	(-0.07, 0.08)	0.86	0.04	(-0.15, 0.23)	0.62	-0.06	(-0.21, 0.09)	0.37
	max	0.00	(-0.25, 0.24)	0.96	-0.08	(-0.23, 0.07)	0.26	0.05	(-0.10, 0.21)	0.48	0.19	(-0.20, 0.59)	0.31	-0.06	(-0.40, 0.28)	0.70
	gm	0.03	(-0.07, 0.13)	0.53	0.00	(-0.08, 0.07)	0.90	0.00	(-0.07, 0.08)	0.89	0.01	(-0.18, 0.19)	0.92	-0.06	(-0.21, 0.08)	0.35

Supplementary Table S6. Association between indoor air pollutants and brain-related indicators after correction for cerebral volume (Model 4)(Continued)

Variable		Cerebral cortex thickness						Subcortical volume								
		Left hemisphere insular lobe			Right hemisphere insular lobe			Left hemisphere nucleus accumbens			Right hemisphere nucleus accumbens			Left hemisphere amygdaloid body		
		estimate	95%CI	p-value	estimate	95%CI	p-value	estimate	95%CI	p-value	estimate	95%CI	p-value	estimate	95%CI	p-value
$PM_{2.5}$	mean	-0.08	(-0.35, 0.19)	0.54	-0.14	(-0.57, 0.29)	0.49	53.78	(-37.67, 145.24)	0.22	-32.93	(-159.22, 93.37)	0.57	-272.09	(-449.34, -94.85)	**0.01**
	gm	0.14	(-0.10, 0.39)	0.22	0.09	(-0.33, 0.50)	0.65	99.14	(35.24, 163.05)	0.01	78.43	(-31.24, 188.10)	0.14	-57.85	(-304.20, 188.50)	0.61
PM_{10}	mean	0.15	(-0.14, 0.45)	0.26	0.17	(-0.31, 0.66)	0.44	47.77	(-59.07, 154.62)	0.34	111.21	(-11.15, 233.58)	0.07	51.12	(-242.12, 344.37)	0.71
	gm	-0.05	(-0.30, 0.20)	0.65	-0.19	(-0.57, 0.18)	0.28	40.06	(-45.09, 125.21)	0.32	-25.82	(-140.75, 89.12)	0.63	-157.16	(-366.25, 51.93)	0.12
CO_2	mean	0.00	(-0.19, 0.20)	0.98	0.20	(-0.09, 0.48)	0.15	-1.65	(-72.60, 69.29)	0.96	-2.56	(-94.58, 89.46)	0.95	-45.86	(-230.18, 138.46)	0.59
	max	0.17	(-0.24, 0.58)	0.38	0.52	(-0.06, 1.11)	0.07	-9.14	(-163.73, 145.45)	0.90	55.18	(-141.71, 252.07)	0.55	15.60	(-392.32, 423.52)	0.93
	gm	-0.04	(-0.23, 0.14)	0.62	0.11	(-0.18, 0.41)	0.41	2.75	(-65.61, 71.12)	0.93	-13.10	(-101.33, 75.13)	0.75	-69.77	(-243.33, 103.78)	0.39

Supplementary Table S6. Association between indoor air pollutants and brain-related indicators after correction for cerebral volume (Model 4)(Continued)

Variable		Subcortical volume														
		Right hemisphere amygdaloid body			Left hemisphere hippocampus			Right hemisphere hippocampus			Left hemisphere globus pallidus			Right hemisphere globus pallidus		
		estimate	95%CI	p-value	estimate	95%CI	p-value	estimate	95%CI	p-value	estimate	95%CI	p-value	estimate	95%CI	p-value
$PM_{2.5}$	mean	-276.69	(-463.52, -89.86)	**0.01**	-473.12	(-990.18, 43.93)	0.07	-516.53	(-971.50, -61.56)	**0.03**	117.18	(-184.39, 418.76)	0.41	159.18	(-137.57, 455.94)	0.26
	gm	-37.67	(-294.54, 219.20)	0.75	52.18	(-535.02, 639.39)	0.85	33.36	(-523.34, 590.07)	0.90	235.36	(-13.47, 484.19)	0.06	192.95	(-78.29, 464.19)	0.14
PM_{10}	mean	-28.98	(-333.85, 275.89)	0.84	306.87	(-354.86, 968.59)	0.33	279.32	(-349.91, 908.55)	0.35	294.11	(7.34, 580.89)	0.05	286.09	(-10.96, 583.14)	0.06
	gm	-194.09	(-397.00, 8.82)	0.06	-135.32	(-684.61, 413.96)	0.60	-100.84	(-624.01, 422.33)	0.68	126.57	(-142.37, 395.52)	0.32	138.51	(-131.91, 408.93)	0.28
CO_2	mean	-67.81	(-255.35, 119.73)	0.44	-253.63	(-656.73, 149.46)	0.19	-244.90	(-625.16, 135.36)	0.18	-59.39	(-279.55, 160.77)	0.56	-42.18	(-267.61, 183.24)	0.69
	max	123.15	(-289.85, 536.16)	0.52	70.74	(-889.48, 1030.96)	0.87	-266.37	(-1157.41, 624.67)	0.52	-205.55	(-672.28, 261.18)	0.35	132.78	(-354.15, 619.70)	0.56
	gm	-98.33	(-271.54, 74.87)	0.23	-300.49	(-669.02, 68.04)	0.10	-312.98	(-649.71, 23.75)	0.07	-56.25	(-268.60, 156.10)	0.57	-24.00	(-242.52, 194.53)	0.81

Supplementary Table S6. Association between indoor air pollutants and brain-related indicators after correction for cerebral volume (Model 4)(Continued)

Variable		Subcortical volume																	
		Left hemisphere putamen			Right hemisphere putamen			Left hemisphere caudate nucleus			Right hemisphere caudate nucleus			Left hemisphere thalamus			Right hemisphere thalamus		
		estimate	95%CI	p-value	estimate	95%CI	p-value	estimate	95%CI	p-value	estimate	95%CI	p-value	estimate	95%CI	p-value	estimate	95%CI	p-value
$PM_{2.5}$	mean	-65.22	(-682.84, 552.40)	0.82	313.83	(-769.02, 1396.67)	0.53	92.65	(-443.52, 628.81)	0.71	91.29	(-395.81, 578.39)	0.69	-296.32	(-1261.78, 669.13)	0.51	-297.55	(-883.36, 288.27)	0.28
	gm	212.06	(-361.08, 785.21)	0.43	692.06	(-245.71, 1629.82)	0.13	305.67	(-163.84, 775.18)	0.18	100.79	(-363.70, 565.28)	0.64	217.07	(-715.14, 1149.27)	0.62	-0.80	(-595.85, 594.25)	1.00
PM_{10}	mean	599.08	(39.65, 1158.52)	0.04	866.53	(-224.91, 1957.98)	0.11	248.54	(-337.03, 834.11)	0.37	211.49	(-324.14, 747.13)	0.40	190.46	(-919.20, 1300.12)	0.71	-19.70	(-723.63, 684.23)	0.95
	gm	-121.32	(-676.01, 433.36)	0.64	301.72	(-676.99, 1280.42)	0.51	127.98	(-353.10, 609.06)	0.57	-0.27	(-445.51, 444.98)	1.00	-119.03	(-1010.22, 772.15)	0.77	-102.91	(-662.07, 456.25)	0.69
CO_2	mean	122.35	(-313.08, 557.78)	0.55	-302.25	(-1065.20, 460.70)	0.40	-200.40	(-560.85, 160.05)	0.24	-154.25	(-489.20, 180.71)	0.33	-272.68	(-954.07, 408.71)	0.39	-208.05	(-629.22, 213.11)	0.30
	max	250.75	(-700.94, 1202.45)	0.57	-387.78	(-2093.36, 1317.79)	0.62	-198.81	(-1031.28, 633.65)	0.61	136.19	(-625.69, 898.08)	0.70	-73.48	(-1616.42, 1469.46)	0.92	-162.53	(-1128.21, 803.15)	0.72
	gm	71.53	(-353.36, 496.42)	0.72	-277.98	(-1015.97, 460.01)	0.42	-189.54	(-537.98, 158.89)	0.25	-136.20	(-461.80, 189.39)	0.37	-286.35	(-938.26, 365.56)	0.35	-238.19	(-633.93, 157.55)	0.21

Supplementary Table S7. Summary Statistics of Magnetic Resonance Imaging Variables Using Hierarchical Clustering Groups

	Variable	Before cerebral volume correction						After cerebral volume correction							
		mean (min–max)			median (Q1–Q3)			mean (min–max)				median (Q1–Q3)			
		Group A	Group B	Group C	Group A	Group B	Group C	Group A	Group B	Group C	Group D	Group A	Group B	Group C	Group D
Cerebral surface area (㎟)	Left hemisphere	-0.08<br>(1.42–1.79)	0.64<br>(-0.45–1.34)	-1.43<br>(-1.7–-1.27)	-0.20<br>(-0.51–0.3)	0.75<br>(0.49–0.98)	-1.37<br>(-1.51–1.29)	-1.24<br>(-2.42–0.44)	0.28<br>(-0.8–1.29)	-0.41<br>(-1.64–0.74)	0.69<br>(-0.33–1.82)	-0.87<br>(-1.65–0.65)	0.14<br>(-0.14–0.89)	-0.20<br>(-1.22–0.28)	0.63<br>(0.07–1.25)
	Right hemisphere	-0.04<br>(-1.4–1.84)	0.62<br>(-0.39–1.35)	-1.45<br>(-1.63–1.29)	-0.02<br>(-0.39–0.31)	0.75<br>(0.39–0.97)	-1.45<br>(-1.51–1.38)	-1.47<br>(-2.23–1.09)	0.25<br>(-0.79–1.71)	-0.25<br>(-1.5–0.94)	0.73<br>(-0.05–2.07)	-1.10<br>(-1.66–1.09)	0.16<br>(-0.06–0.56)	-0.16<br>(-0.99–0.46)	0.45<br>(0.17–1.01)
Cerebral cortex thickness (㎜)	Overall average	-0.93<br>(-2.16–0.1)	0.46<br>(0.41–1.41)	0.94<br>(0.72–1.5)	-1.10<br>(-1.16–-0.21)	0.40<br>(0.15–0.98)	0.80<br>(0.73–1.01)	0.57<br>(0.42–0.72)	0.66<br>(-0.25–1.68)	-1.37<br>(-1.75–-0.82)	-0.52<br>(-1.19–-0.24)	0.56<br>(0.49–0.64)	0.52<br>(0.06–1.2)	-1.41<br>(-1.52–1.33)	-0.33<br>(-0.6–-0.26)
	Left hemisphere average	-0.91<br>(-2.36–0.05)	0.39<br>(-0.32–1.3)	1.08<br>(0.56–1.64)	-0.92<br>(-1.19–0.47)	0.46<br>(-0.24–0.84)	1.05<br>(0.57–1.56)	0.42<br>(0.15–0.77)	0.71<br>(-0.13–1.47)	-1.34<br>(-1.85–-0.76)	-0.60<br>(-1.24–-0.18)	0.33<br>(0.24–0.55)	0.94<br>(0.15–1.25)	-1.52<br>(-1.57–1.02)	-0.48<br>(-0.77–0.31)
	Right hemisphere average	-0.89<br>(-1.8–0.14)	0.51<br>(-0.49–1.9)	0.74<br>(0–1.26)	-1.05<br>(-1.22–0.27)	0.49<br>(-0.13–1.04)	0.84<br>(0.62–0.95)	0.68<br>(0.63–0.75)	0.56<br>(-0.35–2.05)	-1.31<br>(-1.56–-0.83)	-0.42<br>(-1.08–0.1)	0.67<br>(0.65–0.71)	0.45<br>(-0.15–1.16)	-1.44<br>(-1.56–1.17)	-0.36<br>(-0.59–0.19)
	Left hemisphere frontal lobe	-0.56<br>(-2.11–1.11)	0.13<br>(-0.87–1.4)	0.93<br>(-0.06–1.74)	-0.67<br>(-1.14–0.51)	0.10<br>(-0.58–0.77)	1.02<br>(0.54–1.41)	0.00<br>(-0.57–0.49)	0.53<br>(-0.72–1.86)	-1.19<br>(-1.86–-0.48)	0.02<br>(-0.77–1.16)	0.08<br>(-0.24–0.29)	0.34<br>(-0.15–1.34)	-1.19<br>(-1.54–-0.48)	-0.16<br>(-0.76–0.62)
	Right hemisphere frontal lobe	-0.59<br>(-1.74–0.64)	0.27<br>(-1.06–2.16)	0.65<br>(-0.09–1.51)	-0.51<br>(-1.2–0.15)	0.05<br>(-0.44–0.92)	0.58<br>(-0.07–1.3)	0.33<br>(-0.35–1.1)	0.42<br>(-0.97–2.39)	-1.18<br>(-1.54–-0.52)	0.07<br>(-0.54–0.63)	0.26<br>(-0.05–0.68)	-0.04<br>(-0.28–1.23)	-1.39<br>(-1.41–-1.02)	0.08<br>(-0.2–0.36)
	Left hemisphere parietal lobe	-0.87<br>(-1.84–0.11)	0.57<br>(-0.15–2.48)	0.51<br>(-0.03–1.16)	-0.76<br>(-1.33–0.41)	0.34<br>(0.08–0.62)	0.46<br>(0.09–0.89)	0.20<br>(-0.12–0.71)	0.67<br>(-0.11–2.52)	-1.29<br>(-1.65–-0.39)	-0.37<br>(-0.78–0.01)	-0.01<br>(-0.06–0.35)	0.52<br>(0.1–0.8)	-1.50<br>(-1.64–1.28)	-0.35<br>(-0.62–0.11)
	Right hemisphere parietal lobe	-0.76<br>(-2.15–0.41)	0.58<br>(-0.55–1.73)	0.28<br>(-0.75–1.07)	-1.00<br>(-1.19–0.08)	0.47<br>(-0.05–1.33)	0.39<br>(-0.13–0.8)	0.46<br>(0.06–1.06)	0.50<br>(-0.77–1.74)	-1.15<br>(-2.14–0.08)	-0.28<br>(-1–0.41)	0.27<br>(0.17–0.67)	0.68<br>(-0.2–1.28)	-1.20<br>(-1.24–1.11)	-0.26<br>(-0.76–0.21)
	Left hemisphere temporal lobe	-0.80<br>(-2.27–0.06)	0.21<br>(-0.85–1.04)	1.27<br>(1.03–1.54)	-0.82<br>(-1.08–-0.19)	0.30<br>(-0.34–0.83)	1.25<br>(1.06–1.46)	0.93<br>(0.8–1.17)	0.49<br>(-0.3–1.41)	-1.15<br>(-1.71–0.17)	-0.62<br>(-1.71–0.02)	0.84<br>(0.82–1)	0.38<br>(-0.18–1.14)	-1.50<br>(-1.59–-0.8)	-0.38<br>(-0.76–-0.25)
	Right hemisphere temporal lobe	-0.88<br>(-2.03–0.28)	0.38<br>(-0.53–1.4)	1.03<br>(0.65–1.52)	-1.00<br>(-1.39–-0.28)	0.44<br>(-0.32–0.9)	0.96<br>(0.86–1.13)	1.06<br>(0.83–1.36)	0.45<br>(-0.61–1.57)	-1.29<br>(-1.78–0.16)	-0.44<br>(-1.02–0.24)	1.00<br>(0.91–1.18)	0.45<br>(0–0.77)	-1.50<br>(-1.53–1.47)	-0.48<br>(-0.77–0.14)
	Left hemisphere occipital lobe	-0.66<br>(-2.54–0.75)	0.31<br>(-0.68–1.62)	0.71<br>(0.39–1.25)	-1.03<br>(-1.24–0.65)	0.38<br>(0–0.47)	0.59<br>(0.4–0.89)	0.86<br>(0.29–1.68)	0.32<br>(-0.59–1.01)	-0.33<br>(-1.15–0.85)	-1.13<br>(-2.59–0.61)	0.61<br>(0.45–1.15)	0.44<br>(0.02–0.68)	-0.82<br>(-1.06–0.55)	-1.27<br>(-1.7–-0.69)
	Right hemisphere occipital lobe	-0.78<br>(-2.22–0.3)	0.50<br>(-1.03–2.27)	0.50<br>(0.04–1.16)	-0.67<br>(-0.98–-0.6)	0.31<br>(-0.08–1.13)	0.41<br>(0.14–0.77)	1.16<br>(0.68–1.59)	0.32<br>(-1.07–2.26)	-0.84<br>(-1.01–0.68)	-0.69<br>(-2.22–0.31)	1.21<br>(0.94–1.4)	0.13<br>(-0.06–0.47)	-0.80<br>(-1–-0.69)	-0.43<br>(-0.97–0.16)
	Left hemisphere cingulate gyrus	-0.82<br>(-1.73–-0.13)	0.18<br>(-0.6–1.4)	1.38<br>(1.07–1.91)	-0.97<br>(-1.27–-0.17)	-0.18<br>(-0.36–0.78)	1.27<br>(1.21–1.44)	1.14<br>(0.83–1.56)	0.46<br>(-0.28–1.33)	-0.89<br>(-1.44–0.38)	-1.00<br>(-2.01–0.24)	1.02<br>(0.93–1.29)	0.58<br>(-0.07–0.97)	-1.30<br>(-1.3–-0.77)	-0.87<br>(-1.32–0.55)
	Right hemisphere cingulate gyrus	-0.43<br>(-1.93–1.15)	0.05<br>(-1.21–1.16)	0.85<br>(-0.23–2.07)	-0.30<br>(-1.09–0.15)	0.06<br>(-0.24–0.65)	0.77<br>(0.34–1.27)	0.45<br>(0.31–0.66)	0.28<br>(-0.93–1.71)	0.30<br>(-0.41–1.28)	-1.48<br>(-2.14–0.35)	0.38<br>(0.35–0.52)	0.21<br>(-0.4–1.71)	0.36<br>(-0.1–0.37)	-1.71<br>(-1.96–1.23)
	Left hemisphere insular lobe	-0.66<br>(-1.62–0.75)	0.18<br>(-0.57–1.17)	1.02<br>(0.17–2.21)	-1.07<br>(-1.42–0.36)	0.00<br>(-0.42–0.83)	0.85<br>(0.6–1.28)	0.26<br>(-0.1–0.59)	0.53<br>(-0.29–1.83)	-1.27<br>(-2.02–-0.5)	-0.08<br>(-1.49–0.43)	0.28<br>(0.09–0.44)	0.10<br>(-0.03–1.23)	-1.21<br>(-1.68–0.93)	0.38<br>(-0.1–0.4)
	Right hemisphere insular lobe	-0.54<br>(-2.5–0.25)	0.37<br>(-0.75–1.69)	0.30<br>(-1.84–1.31)	-0.58<br>(-0.65–0.04)	0.30<br>(-0.17–0.66)	0.86<br>(-0.07–1.23)	0.45<br>(-0.23–1.12)	0.33<br>(-1.93–1.75)	-0.87<br>(-2.45–0.01)	-0.15<br>(-0.95–0.24)	0.47<br>(0.12–0.79)	0.38<br>(-0.08–0.94)	-0.70<br>(-0.75–0.46)	0.05<br>(-0.22–0.12)
	Left hemisphere nucleus accumbens	-0.61<br>(-1.37–-0.06)	0.41<br>(-1.07–1.63)	0.35<br>(-1.04–1.93)	-0.69<br>(-0.81–-0.24)	0.23<br>(-0.42–1.47)	0.25<br>(-0.67–1.27)	0.08<br>(-1.05–1.03)	0.48<br>(-1.07–1.91)	-0.59<br>(-1.35–-0.05)	-0.63<br>(-0.89–0.09)	0.24<br>(-0.4–0.64)	0.23<br>(-0.48–1.5)	-0.63<br>(-0.7–-0.24)	-0.77<br>(-0.84–0.56)
	Right hemisphere	-0.81	0.85 (0.16–1.43)	-0.31	-0.80	0.96	-0.19	-0.41	0.79	-0.75	-0.93	0.05	0.76	-0.74	-0.90

	nucleus accumbens	(-1.96–0.03)		(-1.64–0.79)	(-1.24–0.36)	(0.45–1.43)	(-0.62–0.12)	(-1.47–0.2)	(-0.27–1.64)	(-1.8–0.22)	(-1.52–0.41)	(-0.71–0.12)	(0.52–1.09)	(-1.38–0.05)	(-1.3–-0.54)
	Left hemisphere amygdaloid body	-0.23 (-2.16–0.64)	0.57 (-0.33–1.41)	-0.90 (-2.06–0.88)	-0.17 (-0.29–0.24)	0.64 (0.03–1.12)	-1.22 (-2.03–0.09)	-1.32 (-1.95–0.36)	0.53 (-0.54–2.1)	-0.75 (-2.05–0.08)	0.47 (-0.27–1.18)	-1.65 (-1.8–-1.01)	0.39 (0.22–0.83)	-0.68 (-0.77–-0.35)	0.49 (0.08–0.88)
	Right hemisphere amygdaloid body	-0.37 (-2.33–0.52)	0.69 (0–1.66)	-0.90 (-1.61–0.02)	0.02 (-0.72–0.37)	0.68 (0.14–1.08)	-1.01 (-1.29–-0.62)	-0.70 (-1.46–0.06)	0.51 (-0.4–1.48)	-1.08 (-2.35–0.31)	0.48 (-0.83–1.52)	-0.57 (-1.01–-0.32)	0.49 (0.19–0.78)	-1.23 (-1.37–0.77)	0.62 (0.15–0.95)
Subcortica l volume (㎣)	Left hemisphere hippocampus	-0.15 (-2.07–0.81)	0.54 (-0.64–1.45)	-1.02 (-2.55–0.26)	0.05 (-0.65–0.42)	0.44 (0.14–1.13)	-0.89 (-1.55–0.36)	-1.42 (-2.61–0.64)	0.48 (-0.41–1.68)	-0.51 (-2–0.37)	0.39 (-0.61–1.46)	-1.01 (-1.81–-0.83)	0.50 (0.15–0.68)	-0.30 (-0.87–0.24)	0.36 (-0.07–0.82)
	Right hemisphere hippocampus	-0.11 (-2.26–0.58)	0.44 (-0.6–1.78)	-0.87 (-2.3–0.6)	0.16 (-0.32–0.46)	0.35 (-0.03–1.02)	-0.90 (-1.67–-0.11)	-1.32 (-2.25–-0.75)	0.41 (-0.85–1.92)	-0.51 (-2.19–0.24)	0.51 (-0.19–1.4)	-0.97 (-1.61–-0.86)	0.25 (-0.03–0.64)	-0.10 (-0.63–0.12)	0.42 (0.13–0.81)
	Left hemisphere globus pallidus	-0.46 (-1.49–1.27)	0.68 (-0.67–1.21)	-0.68 (-1.35–0.68)	-0.81 (-1.19–0.27)	0.84 (0.56–1.04)	-1.01 (01.18–-0.51)	-0.23 (-0.68–0.5)	0.55 (-0.5–2.78)	-0.37 (-1.17–0.99)	-0.86 (-1.8–0.56)	-0.52 (-0.6–-0.01)	0.29 (-0.01–0.85)	-0.76 (-0.77–0.15)	-1.10 (-1.35–-0.62)
	Right hemisphere globus pallidus	-0.28 (-1.68–1.17)	0.59 (-0.47–1.41)	-0.84 (-1.84–0.76)	-0.47 (-0.81–0.26)	0.55 (0.25–1.14)	-1.13 (-1.38–0.59)	-0.96 (-1.4–-0.52)	0.50 (-0.57–2.28)	-0.35 (-0.48–-0.03)	-0.21 (-1.99–1.54)	-0.97 (-1.18–-0.74)	0.27 (-0.33–1.23)	-0.39 (-0.43–-0.39)	-0.19 (-0.93–0.53)
	Left hemisphere putamen	-0.82 (-1.56–0.97)	0.82 (-0.16–1.78)	-0.20 (-0.58–0.37)	-1.10 (-1.38–-0.34)	0.87 (0.61–1.12)	-0.30 (-0.52–0.02)	0.53 (0.15–1.02)	0.62 (-0.08–2.07)	-0.86 (-1.86–0.11)	-1.03 (-1.9–-0.35)	0.43 (0.29–0.73)	0.43 (0.16–0.71)	-0.75 (-1.3–-0.29)	-0.95 (-1.34–0.64)
	Right hemisphere putamen	-0.67 (-1.83–0.93)	0.47 (-0.12–1.13)	0.34 (-0.92–2.76)	-0.80 (-0.99–-0.3)	0.31 (0.08–0.87)	-0.25 (-0.55–0.64)	-0.06 (-0.72–0.84)	0.56 (-0.11–3.07)	-0.51 (-1.21–0.79)	-0.85 (-1.81–0)	-0.29 (-0.5–0.27)	0.20 (-0.03–0.87)	-0.64 (-1.05–0.47)	-0.79 (-1.17–0.46)
	Left hemisphere caudate nucleus	-0.55 (-1.97–1.48)	0.65 (-0.27–1.24)	-0.40 (-1.29–1.09)	-0.89 (-1.08–0.12)	0.67 (0.43–1)	-0.69 (-0.98–-0.1)	0.14 (-0.73–1.23)	0.45 (-0.52–2.39)	-0.24 (-1.88–1.23)	-1.05 (-1.64–-0.3)	-0.07 (-0.4–0.58)	0.36 (-0.11–0.89)	0.00 (-0.62–0.05)	-1.13 (-1.38–-0.8)
	Right hemisphere caudate nucleus	-0.45 (-1.43–1.4)	0.69 (0.15–1.7)	-0.70 (-1.94–0.38)	-0.92 (-1.08–0.34)	0.63 (0.39–0.89)	-0.62 (-1.31–0.01)	0.05 (-1.57–1.12)	0.43 (-0.87–2.04)	-0.06 (-1.26–0.33)	-1.14 (-1.59–0.28)	0.59 (-0.49–0.85)	0.05 (-0.08–0.97)	0.18 (0.15–0.29)	-1.34 (-1.49–0.99)
	Left hemisphere thalamus	-0.28 (-1.85–0.74)	0.54 (-0.88–1.74)	-0.72 (-1.64–0.42)	-0.17 (-0.51–0.74)	0.72 (-0.21–1.3)	-0.83 (-1.23–-0.33)	-0.52 (-1.51–0.23)	0.37 (-1.83–1.7)	-0.90 (-1.22–0.45)	0.50 (-0.42–1.04)	-0.29 (-0.9–-0.03)	0.33 (-0.22–1.27)	-0.95 (-1.12–-0.74)	0.68 (0.4–0.77)
	Right hemisphere thalamus	-0.15 (-1.94–0.88)	0.56 (-0.86–1.89)	-1.05 (-1.35–-0.8)	-0.01 (-0.22–0.38)	0.22 (-0.01–1.23)	-1.03 (-1.12–-0.97)	-0.48 (-0.72–0.07)	0.23 (-1.08–2.16)	-0.89 (-1.55–0.35)	0.84 (0.08–1.46)	-0.65 (-0.69–0.36)	0.16 (-0.71–0.8)	-1.21 (-1.34–0.69)	0.92 (0.56–1.2)

Supplementary Table S8. Summary Statistics of Indoor Air Pollutants Using Hierarchical Clustering Groups

variable		Before cerebral volume correction						After cerebral volume correction							
		mean (min–max)			median (Q1–Q3)			mean (min–max)				median (Q1–Q3)			
		Group A	Group B	Group C	Group A	Group B	Group C	Group A	Group B	Group C	Group D	Group A	Group B	Group C	Group D
$PM_{2.5}$ (㎍/㎥)	mean	15.58 (9.74–24.9)	19.67 (5.41–52.8)	18.03 (13.18–27.5)	14.20 (13.72–15.3)	17.16 (12.95–22.6)	15.71 (14.00–19.7)	18.39 (14.27–23.7)	19.42 (5.41–52.)	15.68 (13.72–20.6)	15.46 (9.74–24.9)	17.14 (15.71–20.4)	15.26 (13.01–22.0)	14.65 (14.02–15.3)	13.56 (12.12–16.8)
	gmean	12.95 (7.12–20.4)	15.72 (4.25–35.8)	13.45 (10.8–16.2)	12.57 (12.06-12.6)	14.56 (9.68–20.0)	13.36 (11.66–15.2)	15.95 (11.95–21.1)	14.83 (4.25–35.8)	13.34 (12.15–16.6)	12.47 (7.12–20.4)	14.77 (13.36–17.9)	12.82 (9.98–16.6)	12.58 (12.57–12.6)	11.15 (9.47–14.1)
	min	2.68 (1.22–4.5)	2.97 (1.00–6.2)	2.61 (1.38–3.3)	2.59 (2.11–3.1)	2.16 (1.08–4.6)	2.86 (2.41–3.0)	3.57 (2.75–4.6)	2.68 (1.00–6.2)	2.76 (1.22–4.5)	2.58 (1.69–3.3)	3.35 (3.05–3.9)	1.90 (1.12–3.8)	2.59 (2.55–2.8)	2.64 (2.01–3.2)
	max	81.56 (38.32–196.)	72.43 (19.2–153.5)	75.61 (49.2–127.1)	57.64 (39.49–125.8)	65.05 (44.54–81.5)	63.02 (59.02–127.2)	65.08 (62.29–69.1)	75.59 (19.2–153.6)	76.65 (38.3–196.)	87.71 (39.5–127.9)	63.75 (63.0–66.4)	60.90 (44.6–106.0)	51.76 (38.7–57.6)	91.74 (53.1–126.3)
PM_{10} (㎍/㎥)	mean	21.73 (11.66–32.95)	26.11 (8.15–61.7)	23.46 (18.7–32.7)	22.44 (18.31–22.9)	22.69 (19.3–28.9)	21.21 (19.5–25.1)	25.61 (18.7–35.4)	25.29 (8.15–61.7)	22.53 (18.3–27.6)	20.72 (11.7–33.0)	22.65 (20.7–29.0)	20.16 (19.5–27.9)	22.85 (20.8–22.9)	19.14 (14.8–25.0)
	gmean	18.16 (8.44–26.5)	21.09 (6.41–41.5)	17.78 (15.4–19.9)	19.33 (15.9–20.1)	19.29 (14.6–25.8)	17.88 (16.1–19.5)	22.34 (15.4–32.1)	19.54 (6.41–41.5)	19.31 (15.9–22.2)	16.73 (8.4–26.5)	19.43 (17.43–25.)	16.60 (15.1–22.3)	19.33 (18.9–20.1)	15.95 (11.5–21.1)
	min	3.62 (1.84–5.5)	4.00 (1.07–8.0)	3.28 (2.09–4.2)	3.75 (2.32–4.6)	2.39 (1.83–6.8)	3.42 (3.05–3.6)	4.91 (3.37–7.1)	3.49 (1.07–8.0)	3.94 (2.04–5.5)	3.21 (1.84–4.6)	4.20 (3.78–5.6)	2.09 (1.9–4.65)	3.75 (3.37–5.0)	3.19 (2.2–4.2)
	max	113.32 (47.05-264.8)	123.00 (28.5–324.2)	135.55 (64.5–187.)	76.01 (54.5–120.9)	92.92 (59.7–162.8)	145.01 (109.8–170.8)	122.58 (77.7–165.1)	127.68 (28.5–324.2)	103.68 (47.1–264.9)	125.38 (54.5–263.5)	124.86 (101.3-145.0)	108.13 (60.6–176.3)	76.01 (54.3–76.1)	91.79 (60.6–156.5)
CO_2 (ppm)	mean	713.13 (442.3–885.28)	754.46 (431.7–1090.7)	949.71 (584.4–1598.0)	801.99 (597.8–862.4)	717.28 (593.7–960.3)	808.25 (695.8-1062.2)	1040.94 (732.9–1597.9)	747.33 (431.73–1090.68)	651.62 (442.3–878.61)	790.02 (597.82–885.28)	792.00 (762.44–1194.97)	691.18 (584.6–950.02)	600.53 (534.66–801.99)	838.49 (760.39–868.12)
	gmean	683.94 (434.1–834.4)	724.70 (430.5–1062.0)	908.80 (559.7–1511.2)	790.91 (579.9–806.4)	681.70 (583.2–903.4)	782.14 (667.5–1023.5)	998.79 (703.4–1511.2)	716.90 (430.53–1062.03)	633.84 (434.14–834.41)	746.57 (579.91–809.07)	781.78 (742.57–1146.5)	648.67 (566.86–902.44)	588.66 (518.12–793.9)	798.65 (738.16–807.06)
	min	424.24 (295.3–617.1)	471.84 (376.5–628.0)	488.82 (410.3–605.7)	430.01 (363.39–450.34)	443.60 (423.4–517.3)	469.67 (431.2–527.3)	544.58 (438.1–605.7)	458.18 (376.5–628.0)	425.58 (295.3–617.1)	422.57 (363.4–462.0)	589.96 (514.04–597.8)	438.84 (416.0–481.8)	430.01 (353.9–431.6)	432.46 (401.8–453.2)
	max	1328.43 (683.6–1971.6)	1334.58 (551.8–2554.5)	1551.41 (1112.7–2455.0)	1432.74 (982.3–1525.2)	1350.05 (857.4–1692.7)	1319.00 (1264.2–1606.2)	1712.41 (1314.8-2455.0)	1310.38 (551.8–2554.5)	1091.63 (683.6–1434.3)	1624.43 (1432.7–1971.6)	1367.51 (1341.1–1911.3)	1323.25 (867.1–1598.2)	982.26 (932.2–1425.8)	1546.72 (1502.0–1669.1)

Gmean = geometric mean
